# Toward a Mechanistic Modeling of Nitrogen Limitation on Vegetation Dynamics

**DOI:** 10.1371/journal.pone.0037914

**Published:** 2012-05-23

**Authors:** Chonggang Xu, Rosie Fisher, Stan D. Wullschleger, Cathy J. Wilson, Michael Cai, Nate G. McDowell

**Affiliations:** 1 Division of Earth and Environmental Sciences, Los Alamos National Laboratory, Los Alamos, New Mexico, United States of America; 2 National Center for Atmospheric Research, Boulder, Colorado, United States of America; 3 Environmental Sciences Division, Oak Ridge National Laboratory, Oak Ridge, Tennessee, United States of America; 4 Division of Intelligence and Space Research, Los Alamos National Laboratory, Los Alamos, New Mexico, United States of America; DOE Pacific Northwest National Laboratory, United States of America

## Abstract

Nitrogen is a dominant regulator of vegetation dynamics, net primary production, and terrestrial carbon cycles; however, most ecosystem models use a rather simplistic relationship between leaf nitrogen content and photosynthetic capacity. Such an approach does not consider how patterns of nitrogen allocation may change with differences in light intensity, growing-season temperature and CO_2_ concentration. To account for this known variability in nitrogen-photosynthesis relationships, we develop a mechanistic nitrogen allocation model based on a trade-off of nitrogen allocated between growth and storage, and an optimization of nitrogen allocated among light capture, electron transport, carboxylation, and respiration. The developed model is able to predict the acclimation of photosynthetic capacity to changes in CO_2_ concentration, temperature, and radiation when evaluated against published data of *V_c,max_* (maximum carboxylation rate) and *J_max_* (maximum electron transport rate). A sensitivity analysis of the model for herbaceous plants, deciduous and evergreen trees implies that elevated CO_2_ concentrations lead to lower allocation of nitrogen to carboxylation but higher allocation to storage. Higher growing-season temperatures cause lower allocation of nitrogen to carboxylation, due to higher nitrogen requirements for light capture pigments and for storage. Lower levels of radiation have a much stronger effect on allocation of nitrogen to carboxylation for herbaceous plants than for trees, resulting from higher nitrogen requirements for light capture for herbaceous plants. As far as we know, this is the first model of complete nitrogen allocation that simultaneously considers nitrogen allocation to light capture, electron transport, carboxylation, respiration and storage, and the responses of each to altered environmental conditions. We expect this model could potentially improve our confidence in simulations of carbon-nitrogen interactions and the vegetation feedbacks to climate in Earth system models.

## Introduction

Nitrogen limitation is an important regulator of vegetation growth and carbon cycles at local, regional, and global scales [1], [2], [3], [4], [5]. This has been shown in temperate and tropical ecosystems [1], but is especially critical in ecosystems at high latitudes [2], [3]. Most ecosystem models simulate the effect of nitrogen on photosynthesis using a prescribed relationship between leaf nitrogen content and photosynthetic capacity (generally represented by *V_c,max_*; the maximum carboxylation rate) [4], [5]. In reality, however, this relationship may vary with different light, temperature, nitrogen availability, and CO_2_ conditions [6], [7], [8]. Photosynthetic capacity is one of the most important parameters affecting simulated carbon fluxes in many ecosystem models [9], [10]. Using a constant relationship between leaf nitrogen content and photosynthetic capacity can thus reduce the reliability of carbon balance predictions under current and future climates. In order to improve the prediction accuracy of nitrogen limitation on photosynthesis, it is important that we build models that account for key factors contributing to the variability in the relationship between leaf nitrogen and photosynthesis.

Nitrogen is a major constituent of proteins for biological processes (e.g. photosynthesis and respiration) [11] and plants need to balance nitrogen investment in proteins for different biological processes to optimize growth and/or survive under specific environmental conditions [7], [12], [13], [14], [15]. Previous studies have illustrated that the altered nitrogen investment in carboxylation enzymes (mainly ribulose-1,5-bisphosphate carboxylase oxygenase, Rubisco) and in light-capturing proteins of thylakoid (responsible for light capture and electron transport) under different light conditions is one of the key factors contributing to the variability in the relationship between leaf nitrogen and photosynthetic capacity [16]. In this paper, we propose two additional types of nitrogen investment that could impact the nitrogen-photosynthesis relationship: respiratory nitrogen and storage nitrogen. Respiratory nitrogen is defined as the nitrogen invested in mitochondrial respiratory enzymes to generate energy (i.e. ATP) to support growth and tissue maintenance [17], [18]. An ideal definition of storage nitrogen would be the nitrogen stored in plant tissues that is not involved in any metabolic processes or structural components (i.e., cell wall and DNA) [19]; however, it would be extremely difficult to quantify the nitrogen investment for all metabolic processes. To facilitate the development of a relatively simple nitrogen allocation model, in this study, ‘storage nitrogen’ is defined as the total plant nitrogen pool minus the amount of nitrogen used in structural components, photosynthetic and respiratory enzymes. The storage nitrogen is assumed to be mainly used in the synthesis of new plant tissues or metabolic enzymes using photosynthetic products (e.g. glucose). It can persist in the form of inorganic nitrogen, amino acid and proteins [14], [19], [20]. Along with stored carbohydrates, storage nitrogen can thus sustain plant growth and survival under situations of plant tissue losses due to unpredictable disturbances (e.g., herbivory attack and browsing) or reduced soil nitrogen availability due to competition [14], [19], [20], [21].

Previous modeling studies that attempt to estimate nitrogen allocation for key photosynthetic enzymes are encouraging [7], [13], [15], [22], [23]; however, no models have simultaneously considered nitrogen allocation to storage, carboxylation, respiration and light harvesting. Furthermore, previous models have mainly focused on the effects of light conditions on nitrogen allocation, with few of them simultaneously incorporating other important environmental factors such as temperature, CO_2_ and nitrogen fertilization. In this study, we develop a complete nitrogen allocation model that incorporates nitrogen trade-offs between growth and storage, and nitrogen optimization among light capture, electron transport, carboxylation, and respiration. The model is first evaluated against published data of *V_c,max_* and *J_max_* (maximum electron transport rate). Sensitivity tests are then conducted to better characterize nitrogen allocation in response to changing environmental parameters across herbaceous, deciduous and evergreen plant species. We expect that the model could help us better understand photosynthetic acclimation (specifically refer to the changes in photosynthetic capability resulting from changes in nitrogen investment within this paper) and also provide a more mechanistic prediction of nitrogen limitation upon photosynthesis.

## Methods

### Model Description

**Figure 1:**
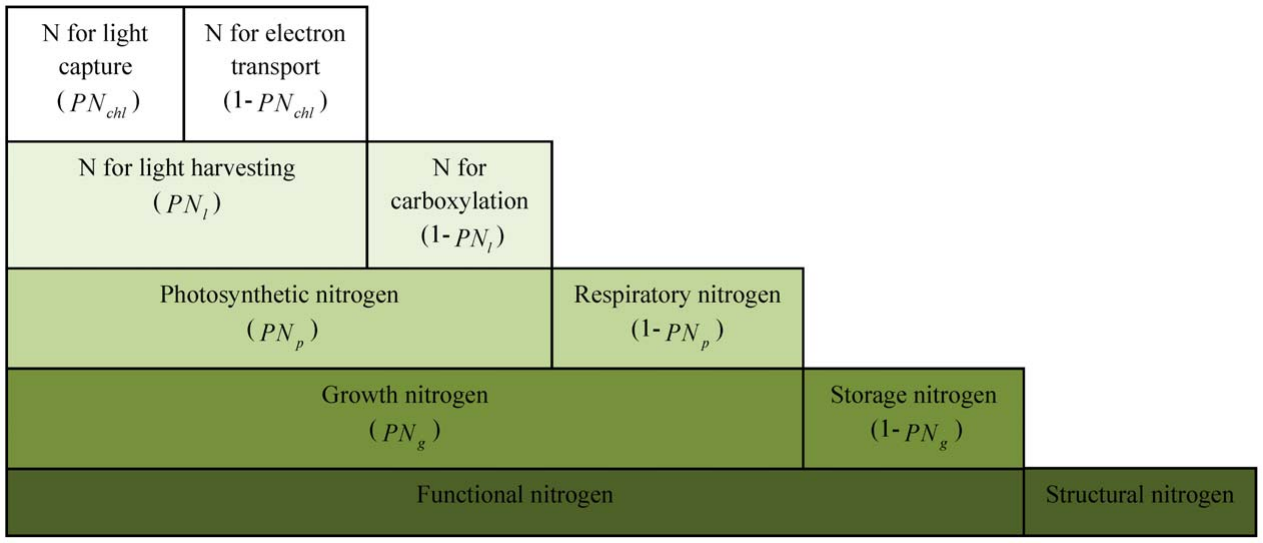
Hierarchical plant functional nitrogen allocation for a leaf layer of a tree. The leaf layer is assigned with a certain amount of plant functional nitrogen (*FNA_a_*) required to support its growth and maintenance. The required plant functional nitrogen includes the functional nitrogen in leaves as well as the functional nitrogen in roots and sapwood, which is used to acquire water and nutrient for photosynthesis and to provide nitrogen for new tissue synthesis using the photosynthetic products. Structural nitrogen is associated with functional nitrogen to build structural components (DNA and cell walls) in tissues of leaves, sapwood and roots. The available functional nitrogen is first divided into growth nitrogen and storage nitrogen. The growth nitrogen is further divided into photosynthetic nitrogen and respiratory nitrogen, with the photosynthetic nitrogen divided into nitrogen for light harvesting and nitrogen for carboxylation (nitrogen in Calvin Cycle enzymes). Finally, nitrogen allocated for light harvesting is divided into nitrogen for light capture (nitrogen in proteins of phosystems I, II and chlorophyll a/b complexes) and nitrogen for electron transport (nitrogen in proteins of thylakoid bioenergetics). The parameter in the parenthesis indicates the proportion of nitrogen invested for its category in the same row.

In our model, plant nitrogen is divided into four pools: structural nitrogen, photosynthetic nitrogen, storage nitrogen and respiratory nitrogen (Figure 1). Structural nitrogen is mainly used to build cell walls and DNA. Because the basic structure of plant cell is similar for different species, the structural nitrogen is set to be fixed at 0.001 (*g* N/*g* biomass), based on data on C∶N ratio from dead wood [24]. Photosynthetic nitrogen is used to build three major classes of proteins∶ proteins for light capture in photosystems I, II and chlorophyll a/b complexes, proteins used as enzymes in the electron transport chain, and proteins for carboxylation in Calvin cycle enzymes. A key assumption of our model is that plants will balance nitrogen allocation to these three classes of proteins to maximize the photosynthesis rate, based on the concept that plants should seek to maximize photosynthetic carbon uptake for a given unit investment of nitrogen [25]. Respiratory nitrogen is located in mitochondrial respiratory enzymes to generate energy (i.e. ATP) required for growth and maintenance [17]. Storage nitrogen is equal to the total size of the nitrogen pool minus structural nitrogen, photosynthetic nitrogen and respiratory nitrogen. A key assumption of the model is that the requirement for storage nitrogen is determined by a parameter that determines how long the storage nitrogen could support the current rate of growth (i.e., the production of new plant tissues and metabolic enzymes) if nitrogen uptake were to cease altogether. We denote this period of time as 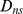 (days). The size of the nitrogen store is affected by the rate of carbon assimilation, nitrogen concentration in new tissues, and species nitrogen use strategy as expressed through 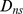. Our definition of storage nitrogen treats all other types of investments not used for structural components, photosynthesis and respiration, such as nitrogen in defense enzymes [12], [14], seed production and enzymes for active nitrogen uptake [26], as storage nitrogen. It would be higher than the actual amount of nitrogen strictly used for storage; however, the inclusion of other type of enzymes in storage nitrogen should not affect the validity of the model because our model will be fitted to the observed *V_c,max_* dataset to estimate the nitrogen storage duration and nitrogen allocation to other types of enzymes could be low [16].

In the model, we define the nitrogen allocated to photosynthesis, storage and respiration collectively as functional nitrogen, which is the total plant nitrogen pool minus the amount of nitrogen used for structural purposes. See Figure 1 for details of nitrogen allocation in this paper and Table S1 for lists of definitions for main model parameters. The ratio of total plant functional nitrogen to the total plant leaf biomass (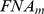, *g* plant functional N/*g* leaf) is an input to the nitrogen allocation model. The above definition of leaf-mass-based plant functional availability substantially simplifies our model by avoiding the complexities of simultaneously tracking multiple pools of functional nitrogen content. 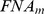 can also be interpreted as the amount of plant functional nitrogen required to support the growth and maintenance of one gram of leaf tissue. The required plant functional nitrogen includes the functional nitrogen in leaves as well as the functional nitrogen in roots and sapwood, which is used to acquire water and nutrient for photosynthesis and to provide nitrogen for new tissue synthesis using the photosynthetic products. Based on 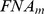, the corresponding leaf-area-based plant functional nitrogen availability (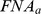, *g* plant functional N/*m* ^2^ leaf) can then be calculated by the multiplication of 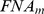 and leaf mass per unit area (*LMA*, g/*m* ^2^). For a constant 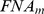, therefore, 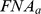 will differ for leaves that have different LMA (e.g. at different locations in the canopy) [27], but the derivation of optimal LMA is beyond the scope of this study.

Our model considers nitrogen allocation within a given leaf layer in the canopy that has a predetermined leaf-area-based plant functional nitrogen availability (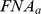) to support its growth and maintenance. The 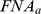 is hierarchically allocated for five major processes (see Figure 1). First, functional nitrogen is allocated between growth and storage based on a plant's strategies of growth and persistence. Second, the growth nitrogen is partitioned into photosynthetic and respiratory nitrogen. Finally, the photosynthetic nitrogen is allocated among light-harvesting, electron transport, and carboxylation.

A complete description of the model is provided in Texts S1, S2, S3. In summary, we impose a series of assumptions on the model to generate the ideal (or optimized) nitrogen distributions. These are *i*) storage is allocated to meet requirements based on multiplication of net photosynthesis rate, nitrogen concentration in new tissues, and nitrogen storage duration (days); *ii*) respiratory nitrogen is equal to the demand implied by the sum of maintenance respiration and growth respiration; *iii*) light capture, electron transport and carboxylation are co-limiting to maximize photosynthesis. The first assumption is built on the inference that higher photosynthesis rates will require more storage nitrogen to support a higher rate of new plant tissue production, and the observation that enhanced photosynthesis rates can be subjected to resource limitation (e.g. nitrogen) or process limitation (e.g. carbon sink limitation) [28]. The storage duration parameter 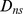 in this assumption determines plants' nitrogen allocation strategy and is reflective of the widely observed trade-off in plant strategies between growth and persistence [12], [29]. The second and third assumptions are about co-limitation of nitrogen allocation among light capture, electron transport, carboxylation and respiration, which are mostly based on the presumption of optimality [25]. The above three assumptions together form a testable hypothesis concerning the function of plant nitrogen allocation under varying environmental conditions.

### Model evaluation

To test if the hypotheses embedded in the nitrogen allocation model are able to predict acclimations of *V_c,max_* and *J_max_* under different environmental conditions (i.e, changes in *V_c,max_* and *J_max_* resulting from changes in nitrogen allocated to carboxylation and electron transport), we evaluated our model against data reported in three independent test cases. For test case 1, *V_c,max_* and *J_max_* (maximum electron transport rate) were measured for one-year old needles from loblolly pine (*Pinus taeda*) trees exposed to ambient (control) and elevated (treatment) CO_2_ concentrations in a Free Air CO_2_ enrichment (FACE) experiment located at the Duke forest [30]. The forest soil is acidic and nutrient-poor soils. For test case 2, *V_c,max_* and *J_max_* were measured for poplar (*Populus tremula*) leaves located at top of canopy (control) and reduced light radiation levels in the canopy (treatment) for a mixed deciduous stand on a sandy loam soil near Ülenurme, Estonia [31]. For test case 3, Japanese plantain (*Plantago asiatica*) was grown in pots from seeds within greenhouses for about 1–2 months at two contrasting temperatures: 30°C (control) and 15°C (treatment) [32]. These studies provide a wide range of environmental conditions to allow testing of the impacts of resource changes on nitrogen allocation, and they each provide the critical data for model fitting purposes, which are *i*) *V_c,max_* and *J_max_* at different levels of leaf nitrogen content, *ii*) LMA (directly or indirectly through other studies), *iii*) photosynthetic active radiation (PAR), and *iv*) growing temperature. See Table 1 for the main model inputs.

**Table 1:** Main model inputs for three test cases.

Test cases	Treatment Time	*PAR* ^1^	[CO_2_] (*ppm*)	Daytime (hours)	DT^2^	*NT* ^2^	*RH* ^3^	*LMA* ^4^	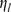 ^5^	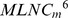
Test case 1 control (Ambient [CO_2_])	8–9 years	1010	370	14	28	23	0.8	85	0.2	0.014
Test case 1 treatment (Elevated [CO_2_])	8–9 years	1010	570	14	28	23	0.8	85	0.2	0.014
Test case 2 control (top of the canopy radiation)	N/A	621	370	17	17	13	0.6	138	0.2	0.0215
Test case 2 treatment (shaded canopy locations)	N/A	621*x*	370	17	17	13	0.6	Reg	0.2	0.0215
Test case 3 control (High temperature)	1–2 months	450	370	14	30	30	0.8	55	0.6	0.03
Test case 3 treatment (Low temperature)	1–2 months	450	370	14	15	15	0.8	66	0.6	0.03

Note 1: ***PAR*** = photosynthetic active radiation for nitrogen allocation among carboxylation, light capture and electron transport (*µmol* photon/*m* ^2^/*s*). Data for test case 1 and 2 is from the 10-km gridded data from the SUNNY model [53] and NCEP/NCAR Reanalysis dataset [54], respectively, averaged for daytime period in July. Data for test case 3 is from the experimental controlled radiation. For shaded canopy locations in test case 2, the radiation level is calculated by multiplying the top of canopy radiation and the relative light (*x*) it receives.2: ***DT*** */* ***NT*** = daytime temperature / nighttime temperature (°C). For test case 1, data are based on average daily minimum and maximum temperature in July from the DAYMET website [55]. For test case 2, data are based on average daily minimum and maximum temperature in July from the NCEP/NCAR Reanalysis dataset [54]. 3: ***RH*** = relative humidity, which is the ratio of the partial pressure of water vapor in the air to the saturated vapor pressure. Data are from the original papers. 4: ***LMA*** = Leaf mass per unit area (*g* /*m* ^2^). For test case 1, *LMA* is calculated based on the mean values of old and new leaves in July [56]. For shaded canopy locations in test case 2, the *LMA* is calculated based on the regression [57]: *y* = 73+65.5*x*, where *x* (0–1) is the radiation of leaf relative to the top of canopy. For the high growing temperature condition in test case 3, data is from Kobayashi et al. [58]. We assume a 20% increase in *LMA* at the low growing temperature given that the area based leaf nitrogen content increased by about 20% at the low growing temperature [32]. 5: 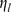 is the proportion of net carbon assimilation allocated to leaf. We set 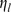 to be 0.2 for test case 1–2 [59] and 0.6 for test case 3 based on fast-growing plants non-woody plants [60]. 6: ***MLNC_m_*** = Mean leaf nitrogen content (*g* N/*g* leaf).

We use a Metropolis-Hastings approach [33], [34] to estimate the two key unknown parameters in the model: the nitrogen storage duration (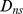) and the proportion of storage nitrogen allocated to leaves (*f*_s_). See Figures S1, S2, S3 for sensitivity analysis of several important unknown parameters in the model. The 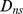 and *f*_s_ are fitted so that the *V_c,max_* determined by carboxylation nitrogen allocation in our model under the control conditions is in a good agreement to the observed *V_c,max_* at different leaf nitrogen concentrations. Refer to Text S4 for a detailed description of the fitting process. In order to test if the model is able to predict the *V_c,max_* at different environmental conditions, we use the model fitted under control conditions to predict *V_c,max_* and *J_max_* for the treatment conditions. See Table 1 for control and treatment conditions for each test case and Table 2 for the estimated parameter values using the Metropolis-Hastings approach.

**Table 2:** Fitted parameter values for test cases 1–3 using Metropolis-Hasting approach.

Parameter	Test case 1	Test case2	Test case 3
Proportion of storage nitrogen allocated to leaf ( 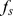 )	0.86 (0.032)	0.54(0.16)	0.52(0.17)
Nitrogen storage duration ( 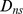 )	85.2 (4.08)	50.4 (16)	3.98(3.09)

**Note:** Values in the parenthesis represent the standard deviation of the fitted parameter value.

Only control condition data are used for fitting the model.

Since the reported values of *V_c,max_* and *J_max_* in different studies can be estimated based on different values of Michealis constants for CO_2_ and O_2_ [i.e., 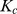 and 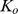 in eqs. (S3.2) and (S3.3) in Text S3] and different temperature dependence functions, we specifically standardize the *V_c,max_* and *J_max_* using the values of 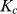 and 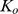 and temperature dependence functions reported by Collatz et al [35]. See Text S5 for specification of temperature dependence functions and Text S6 for details of standardization. For test case three, Hikosaka [32] measured the *V_c,max_* at both 15°C and 30°C. To reduce the effect of measurement temperature on active status of Rubisco [36], we estimate the *V_c,max_* and *J_max_* at 25°C by scaling from that measured at the plant's growing temperature (15 or 30°C) using temperature dependence functions in Text S5.

### Model sensitivity analysis

**Figure 2:**
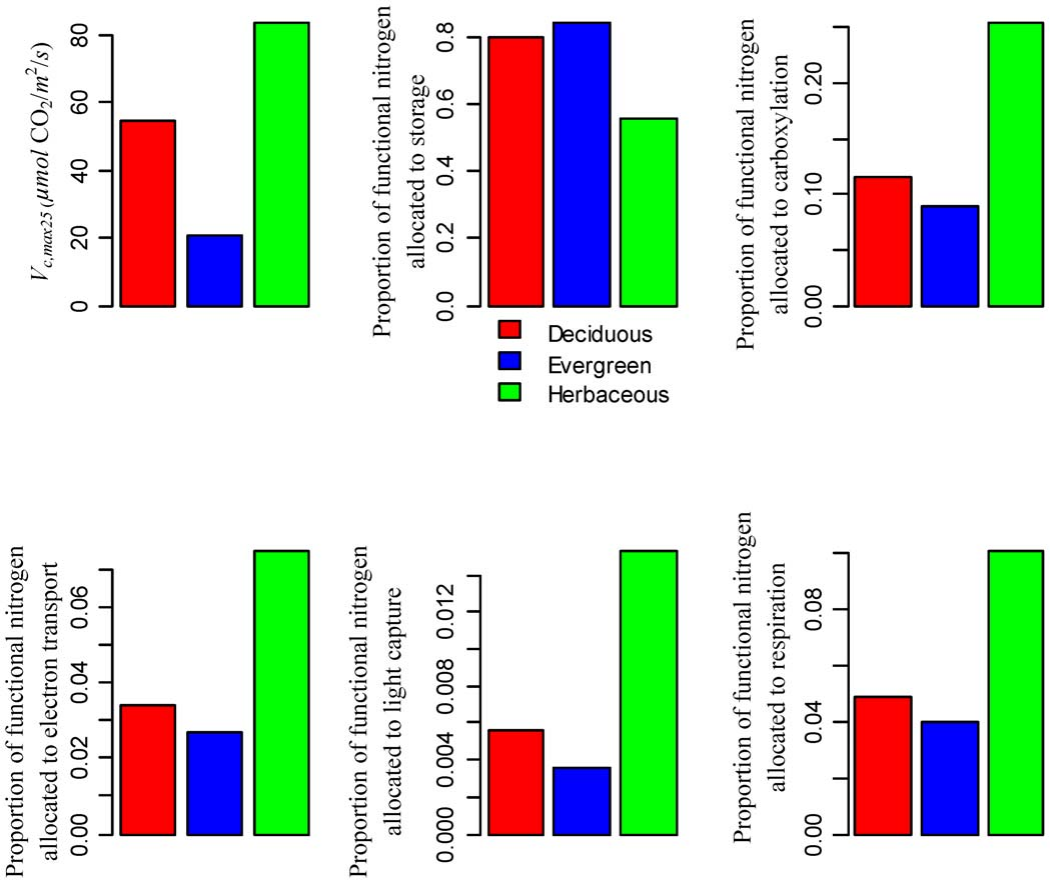
*V_c,max25_* (*V_c,max_* scaled to 25°C) and nitrogen allocation coefficients for deciduous trees, evergreen trees, and herbaceous plants for the control case (temperature = 15°C; radiation = 800 *µmol* photon/*m* ^2^/*s*; CO_2_ = 370 *ppm*) in the sensitivity analysis. The nitrogen allocation coefficients are estimated with the nitrogen allocation model using parameter inputs from Table 3.

To better understand the model prediction of nitrogen allocation responses to changes in growing temperature, radiation and CO_2_ concentration, we conduct a sensitivity analysis for the model with three representative generic species including coniferous and deciduous tree species and an herbaceous species. Based on our model fitting against these three different species (see Table 2), the nitrogen storage duration parameter is set to be 50, 85 and 4 days for deciduous trees, evergreen trees, and herbaceous plants, respectively. Based on these estimates of plant nitrogen storage strategy, we explore the photosynthetic acclimation resulting from modeled changes in nitrogen allocation coefficients to *i*) an increase in growing temperature from 15°C to 20°C, *ii*) an increase in CO_2_ concentration from 370 to 570 *ppm*, and *iii*) photosynthetic active radiation reduction from 800 to 400 *µmol* photon/*m* ^2^/*s*. We use a factorial experimental design to test the effect of above changes in temperature, CO_2_, radiation, and their interactions on photosynthetic acclimation. Since our main focus of this paper is to explore nitrogen allocation acclimation under different environmental conditions, we assume that potential leaf nitrogen and leaf mass per unit area are constant. See Table 3 for detailed model parameter values used in the sensitivity analysis and Figures S4, S5 for some representative fitting of the model for different species. Estimated *V_c,max_* and nitrogen allocation coefficients are shown in Figure 2 for the control condition with a growing temperature of 15°C, a CO_2_ concentration of 370 ppm and a mean photosynthetic active radiation reduction of 800 *µmol* photon/*m* ^2^/*s*.

**Table 3:** Model parameters of deciduous, evergreen trees and herbaceous plants for the sensitivity analysis.

Parameter	Deciduous	Evergreen	Herbaceous
Proportion of storage nitrogen allocated to leaf (*f_s_*)	0.86	0.5	0.5
Nitrogen storage duration (*D_ns_*)	85	50	4
Leaf mass per unit area (LMA; *g*/*m* ^2^)	120	85	60
Leaf nitrogen content (*g* N/*g* biomass)	0.02	0.015	0.03
Proportion of net carbon assimilation allocated to leaf biomass ( 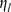 )	0.2	0.2	0.6

## Results

**Figure 3:**
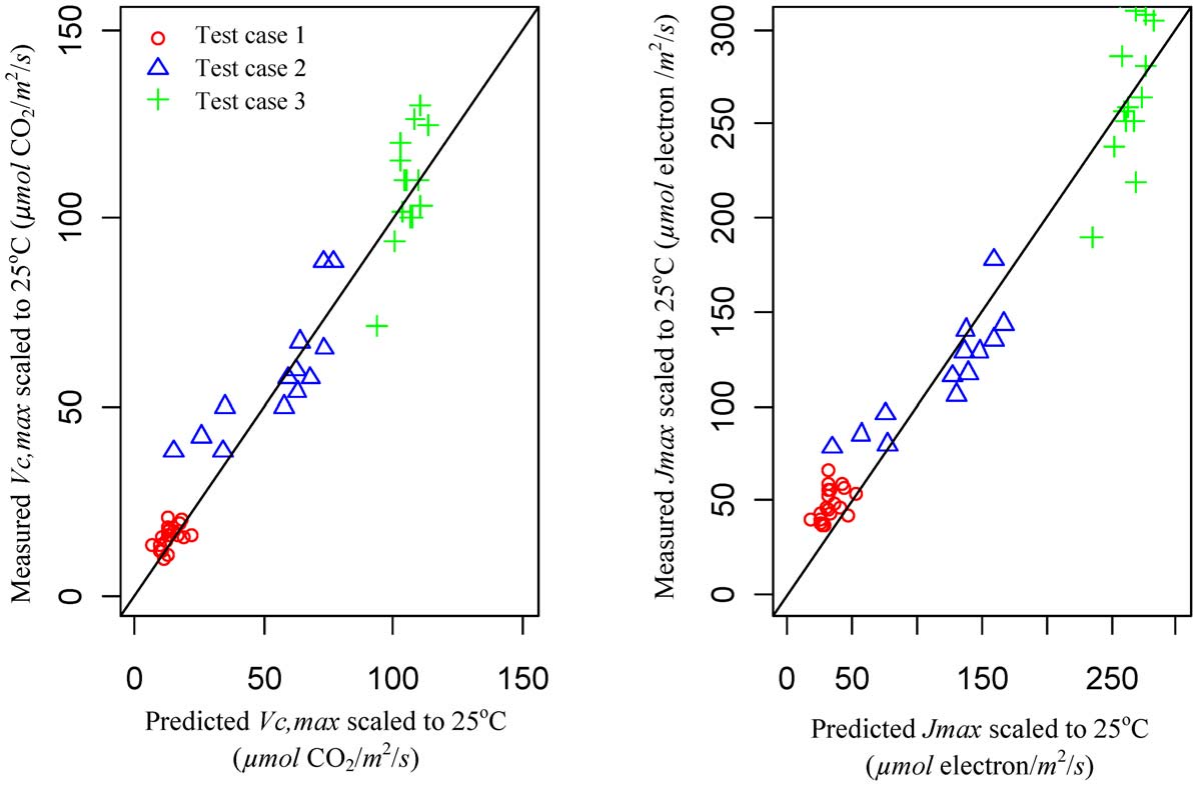
Nitrogen allocation model evaluations. The figure shows the scatter plots of predicted and measured *V_c,max_* and *J_max_* scaled to 25°C (i.e.,*V_c,max25_* and *J_max25_*) at elevated CO_2_ (570 *ppm*, test case one), reduced radiation in canopy (0.1–0.9 of the radiation at the top of canopy, test case two) and reduced growing temperature (15°C, test case three). The model is first calibrated using control data under ambient CO_2_ (370 ppm), radiation at the top of the canopy (621 *µmol* photon/*m^2^/s*), the normal growing temperature (30°C). The fitted model is then used to predict *V_c,max25_* and *J_max25_* under altered environments.

The pooled *R^2^* coefficient for test cases 1–3 between predicted and measured *V_c,max25_* (i.e., *V_c,max_* scaled to 25°C using temperature dependence functions in Text S5) is 0.953 with a root mean square error (RMSE) of 9.09 *µmol* CO_2_/*m* ^2^/*s* (Figure 3). This suggests that the nitrogen allocation model is able to capture the acclimation of *V_c,max25_* (i.e., the change of *V_c,max25_* resulting from an increase or a decrease in amount of nitrogen allocated to Rubisco) reasonably well under elevated CO_2_ concentrations, reduced growing temperature and reduced radiation conditions. Although the calibrated model does not use *J_max_* data under control conditions, it is still able to reproduce the measured *J_max_* scaled to 25°C (i.e., *J_max25_*) under treatment conditions reasonably well (Figure 3). The mean *R^2^* coefficient for test cases 1–3 between predicted and measured *J_max25_* under treatment conditions is 0.943 with a root mean square error (RMSE) of 9.43 *µmol* electron/*m* ^2^/*s*. The equally good agreements between predicted and measured *V_c,max25_* and *J_max25_* (Figure 3) is strong evidence to support our key model assumption of co-limitation of light harvesting, carboxylation, and electron transport.

**Figure 4:**
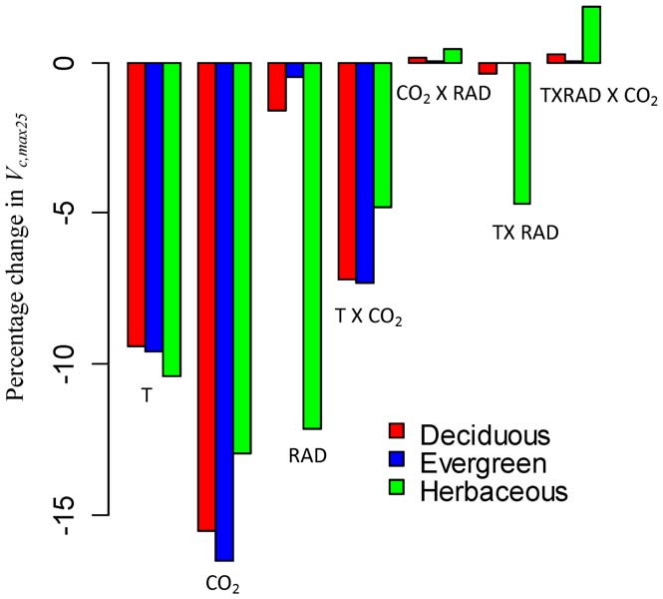
Acclimation of *V_c,max25_* (*V_c, max_* scaled to 25°C) to altered environmental conditions. The nitrogen allocation model is used to quantify the responses of *V_c,max25_* to increased growing temperature (from 15°C to 20°C; labeled as “T” in the figure), increased CO_2_ concentration (from 370 *ppm* to 570 *ppm*; labeled as “CO_2_” in the figure) and reduced radiation (from 800 to 400 *µmol* photon/*m* ^2^/*s*; labeled as “RAD” in the figure) and their interactions for generic deciduous trees, evergreen trees, and herbaceous plants. The change of *V_c,max25_* to environmental conditions in our model results from a decrease or an increase in nitrogen allocation to carboxylation. See Figure 2 for *V_c,max25_* and nitrogen allocation coefficients for the control case. See Table 3 for main input parameters of the model.

**Figure 5:**
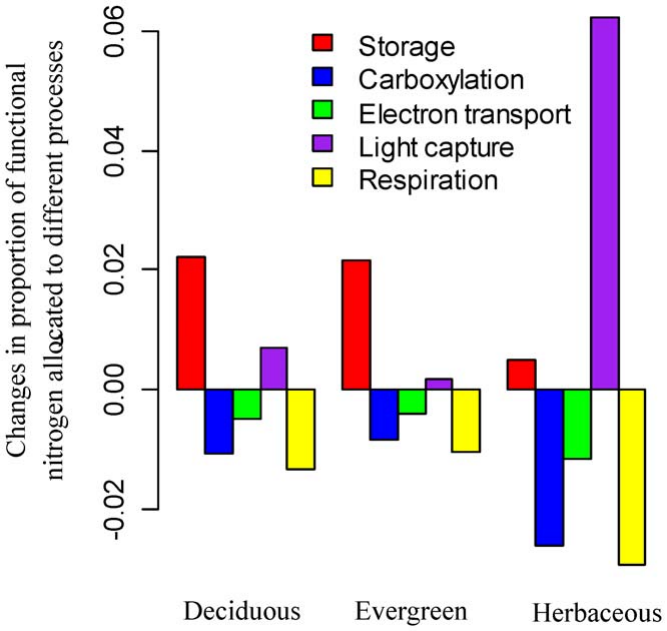
Temperature effects on nitrogen allocation coefficients for deciduous trees, evergreen trees, and herbaceous plants. We explore the effect of increased growing temperature from 15°C to 20°C on the proportions of nitrogen allocated to storage, carboxylation, electron transport, light capture and respiration for a leaf layer with prescribed functional nitrogen availability. Positive values indicate increase in nitrogen allocation while negative values indicate decrease in nitrogen allocation.

The model sensitivity analysis predicts that an increase of growing-season temperature from 15°C to 20°C will down-regulate *V_c,max25_* by about 10% (Figure 4), due to a decrease in nitrogen allocation to carboxylation (Figure 5). Note that “down-regulate” or “up-regulate” in this context refers to the change of *V_c,max25_* resulting from a decrease or an increase in nitrogen allocation to carboxylation (assuming no leaf nitrogen content change). The *V_c,max_ per se* may increase with temperature even with down-regulations of *V_c,max25_*. The main reason for a predicted decrease in nitrogen allocation to carboxylation is that increased temperature enhances enzyme catalytic rates for carboxylation, electron transport and respiration. To achieve the nitrogen allocation balance between growth and storage, our model predicts higher nitrogen allocation to storage but lower nitrogen allocation to carboxylation, electron transport, and respiration (Figure 5). Since light capture is assumed to be unaffected by leaf temperature [37], our model also predicts higher nitrogen allocation to light capture to support the higher rate of carboxylation at a higher temperature (Figure 5). This effect is especially evident for herbaceous plants (Figure 5), due to the fact that herbaceous plants have a relatively high value of *V_c,max25_* in our sensitivity analysis (Figure 2) and thus higher nitrogen allocation to light capture is required to compensate for the larger absolute stimulation of carboxylation by increased temperature.

**Figure 6:**
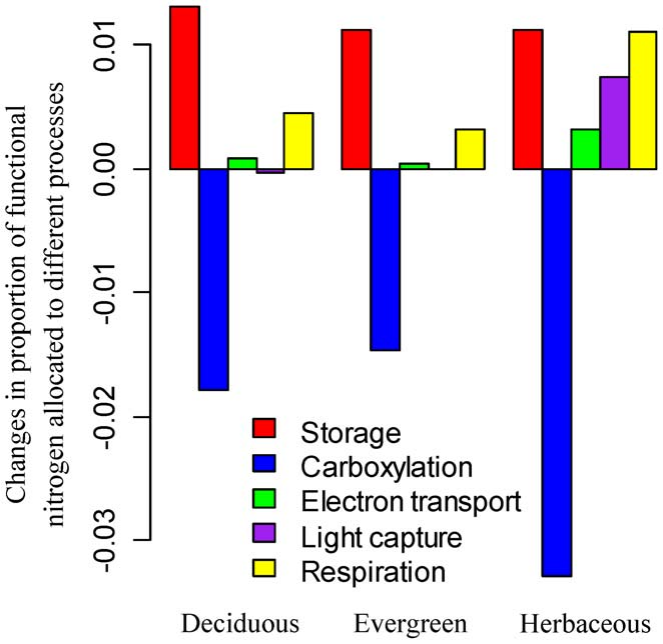
Effects of elevated CO_2_ concentration on nitrogen allocation coefficients for deciduous trees, evergreen trees, and herbaceous plants. We explore the effects of increased CO_2_ concentration from 370 *ppm* to 570 *ppm* on the proportions of nitrogen allocated to storage, carboxylation, electron transport, light capture and respiration for a leaf layer with prescribed functional nitrogen availability. Positive values indicate increase in nitrogen allocation while negative values indicate decrease in nitrogen allocation.

The nitrogen allocation model predicts that an increase in CO_2_ concentration from 370 to 570 *ppm* could down-regulate the *V_c,max25_* by about 15% (Figure 4), due to a decrease in nitrogen allocation to carboxylation (Figure 6). The main reason for predicted lower nitrogen allocation to carboxylation is that elevated CO_2_ concentration enhances substrate concentration for Rubisco and thus leads to higher carboxylation rates for the same amount of Rubisco [see eq. (S3.1) in Text S3]. To achieve nitrogen investment balance between growth and storage, the model predicts higher nitrogen allocation to storage and lower nitrogen allocation to carboxylation (Figure 6). To balance the increased carboxylation rate, the model also predicts a relatively large increase in the allocation of nitrogen to respiration (Figure 6).

**Figure 7:**
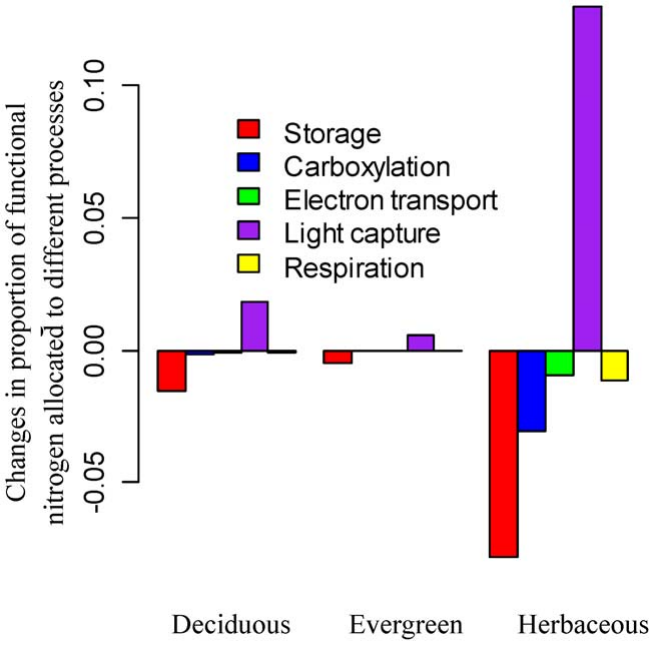
Radiation effects on nitrogen allocation coefficients for deciduous trees, evergreen trees, and herbaceous plants. We explore the reduced radiation from 800 to 400 *µmol* photon/*m* ^2^/*s* on the proportions of nitrogen allocated to storage, carboxylation, electron transport, light capture and respiration for a leaf layer with prescribed functional nitrogen availability. Positive values indicate increase in nitrogen allocation while negative values indicate decrease in nitrogen allocation.

Reducing irradiance from 800 to 400 *µmol* photo/*m* ^2^/*s* within the model has only a small effect (<3%) on acclimation of *V_c,max25_* for deciduous and evergreen trees, but imposes a strong down-regulation (∼10%) of *V_c,max25_* for herbaceous plants (Figure 4). For deciduous and evergreen trees, the model predicts that lower radiation decreases nitrogen allocated to storage (Figure 7), due to the lower storage nitrogen requirement induced by a lower photosynthesis rate. Meanwhile, to compensate for a lower level of radiation, the model predicts an increase in nitrogen allocated to light capture (Figure 7). The combination of increased nitrogen allocation to storage and decreased nitrogen allocation to light capture leads to a slight decrease or no change in nitrogen allocated to carboxylation (Figure 7). Compared with deciduous and evergreen trees, the model predicts a much larger increase in the nitrogen allocation to light capture for herbaceous plants, which leads to much decreased nitrogen allocation to carboxylation (Figure 7) and down-regulation of *V_c,max25_* (Figure 4). This is because herbaceous plants have a relatively high *V_c,max25_* (Figure 2) and thus a much large increase in nitrogen allocation to light capture is required to compensate for the reduction in light intensity so that the light capture rate equal to the Rubisco-limited carboxylation rate (Figure 7).

Our sensitivity analysis shows that there is a strong interaction between temperature and CO_2_ on down-regulation of *V_c,max25_* for deciduous and evergreen trees (Figure 4). This is because the effect of increased CO_2_ concentration on carboxylation will be stronger at a higher temperature with a higher maximum carboxylation rate (see eq. (S3.1) in Text S3). The model also predicts a strong interaction between temperature and radiation on *V_c,max25_* acclimation for herbaceous plants (Figure 4). This is because the model predicts that more nitrogen will be required for light capture with a higher temperature (see Figure 5), leading to lower nitrogen allocation to carboxylation and a greater down-regulation of *V_c,max25_* at a higher growing temperature. This interaction effect is small for deciduous and evergreen trees because the there is little effect of decreased radiation on *V_c,max25_* for deciduous and evergreen trees (Figure 4).

## Discussion

A complete nitrogen allocation model based on a trade-off of nitrogen allocated between growth and storage, and an optimization of nitrogen allocated for light capture, electron transport, carboxylation, and respiration is developed to facilitate a better understanding of nitrogen limitations to photosynthesis. Our three test cases with changes in CO_2_ concentration, temperature, and radiation demonstrate the model's capability to investigate the impact of altered environmental conditions on nitrogen allocation (Figure 3). By predicting nitrogen allocation coefficients under different environmental conditions, this model provides a useful tool toward a mechanistic prediction of photosynthetic acclimation.

Our model results imply that higher growing-season temperature decreases nitrogen investment to carboxylation but increases nitrogen investment to light capture (Figure 5). This is in agreement with field and lab experiment data showing that, when plants were transplanted to lower temperatures, the investment of nitrogen to active Rubisco increases but the investment in chlorophyll decreases for most cold tolerant species [32], [36], [38], [39], [40]. For the arctic, this indicates that the response of plant photosynthesis to temperature increase can be much smaller than that predicted from the Farquhar photosynthesis model [41] (assuming no acclimation in nitrogen allocation). Reich et al. [42] observed that the relationship between photosynthesis and leaf nitrogen is stronger in the arctic than in the tropics. They attributed this to the higher ratio of phosphorus to nitrogen in leaves of arctic plants. Based on our model, an alternative hypothesis is that lower chlorophyll requirements at low temperature in the arctic lead to higher allocation of nitrogen to carboxylation. The higher nitrogen allocation to carboxylation should ultimately lead to a stronger relationship between leaf nitrogen and photosynthetic capacity.

Our model sensitivity analysis predicts a down-regulation of *V_c,max25_* by about 15% with 200 *ppm* CO_2_ enrichment (Figure 4), which is in the range of reported values from empirical studies [43], [44]. Note that the CO_2_ enrichment not only affects the individual leaf level photosynthesis, but also affect the whole plant leaf biomass [45]. Thus, even with the down-regulation of *V_c,max25_*, the CO_2_ enrichment effect of net primary production could be large [45]. The potential improvement with our nitrogen allocation model will be mainly beneficial for leaf-level prediction of photosynthesis; however, it will be also helpful for the whole-plant level prediction of leaf biomass production with improved simulation of photosynthesis.

Our model sensitivity analysis suggests that *V_c,max25_* for deciduous and evergreen trees is much less responsive to radiation reduction compared to herbaceous plants (Figure 4). This is in agreement with reports of a substantial reduction in Rubisco allocation for herbaceous plants with reduced radiation levels [46], but small changes in Rubsico allocation compared to leaf anatomical changes (e.g., LMA) for trees [15], [31]. In our sensitivity analysis we assume that the leaf-area-based functional nitrogen availability does not change with reduced radiation; however, it may decrease in view that leaf-area based nitrogen content might reduce in low radiation environments resulting from decreased LMA [47]. If we feed the model with reduced leaf-area-based functional nitrogen availability, our model reasonably predicts changes of *V_c,max25_* with a gradient of light conditions for test case two (see Figure 3). For a prognostic prediction, the nitrogen allocation model should be coupled with models that predict *LMA* as well as functional nitrogen availability under different environmental conditions.

**Figure 8:**
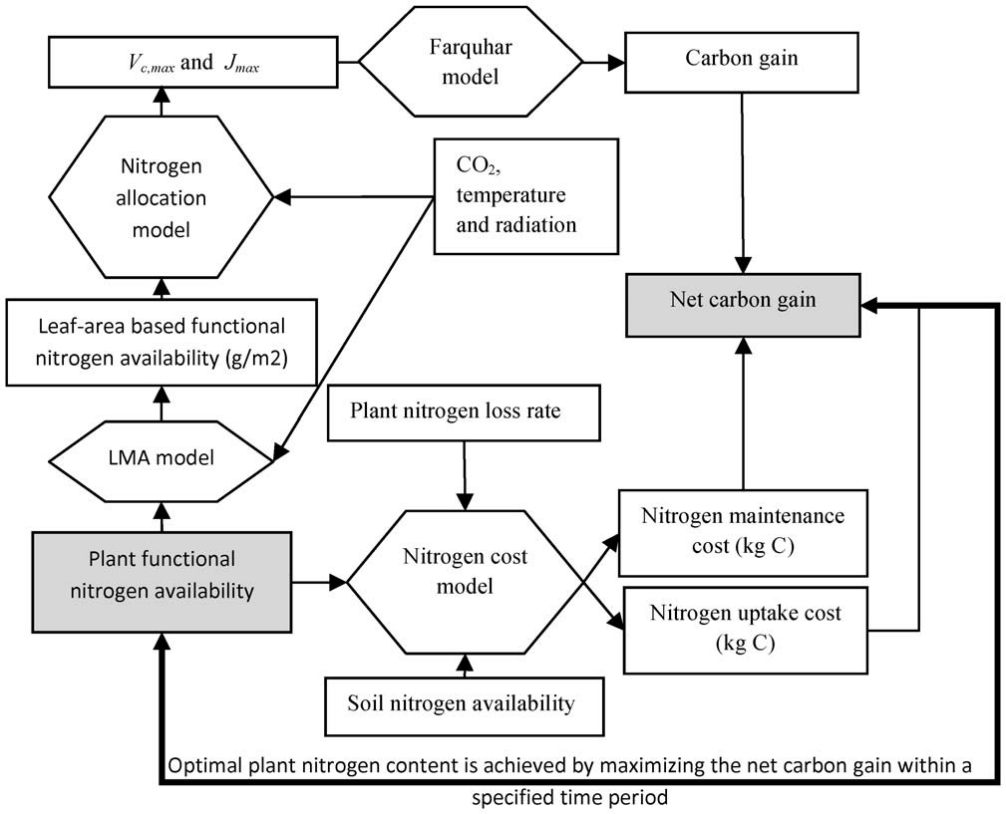
Plant functional nitrogen availability optimization by linking a nitrogen allocation model and a nitrogen cost model. The rectangles represent state variables of interests. The shaded ones represent the target variables to achieve optimization/maximization. The hexagons represent models. Optimal plant functional nitrogen availability is achieved by maximizing the net carbon gain within a specified time period. The net carbon gain is based on the net carbon balance between photosynthesis and carbon cost of plant nitrogen maintenance and uptake. Nitrogen allocation model is used to predict the photosynthesis parameters (*V_c,max_* and *J_max_*) for the Farquhar photosynthesis model.

We propose that the nitrogen allocation model should be useful in ecosystem process models or dynamic global vegetation models to represent plant acclimation processes more mechanistically and to estimate the optimal plant nitrogen content that maximizes net carbon profit (Figure 8). The net carbon profit could be estimated by the gross primary production minus growth respiration, maintenance respiration, and the nitrogen uptake costs. Gross primary production could be determined by the nitrogen allocation model and the Farquhar photosynthesis model [41] integrated over leaf layers. Since the response of LMA to light conditions could have a much stronger effect on photosynthesis than the changes in nitrogen allocation [15], [47], it is important to incorporate a prognostic LMA model to estimate the leaf area given a certain amount of leaf nitrogen content and different light, temperature, and CO_2_ conditions [48]. Nitrogen uptake costs can be estimated based on soil nitrogen availability [49]. Improved simulation of plant nitrogen allocation may be beneficial to ecosystem process and dynamic global vegetation models, but would require testing against data from different geographic locations (arctic, temperate and tropical forests).

Although we have shown that our model is able to predict the acclimation of nitrogen allocation to different environmental conditions reasonably well based on evaluation against *V_c,max_* and *J_max_* data, our model remains based on a set of assumptions concerning the nature of plant optimality, the validity of which need further testing. Currently, there are four important limitations in the model. Firstly, the nitrogen in storage is difficult to measure since stored nitrogen can be present in different forms [20], [50]. Furthermore, Rubisco can function as both carboxylation enzyme or storage nitrogen [51]. Therefore, it is difficult to compare simulation results with observations. For example, our model predicts high nitrogen allocation to storage (∼0.8) in loblolly pine (see Figure S4). This result may be difficult to validate against observed data; however, the high nitrogen allocation to “storage” could be reasonable in view that the proportion of total nitrogen allocated to processes other than light capture and carboxylation for the herbaceous species *Phaseolus vulgaris* is as high as 0.65 [7], [52] and that trees require more storage nitrogen to buffer long-term environmental fluctuations. Note that we consider all enzymes except for those involved in carboxylation, light capture, electron transport and respiration as storage nitrogen.

Second, storage nitrogen in the model is mainly used to produce new plant tissues including both structural and photosynthetic components; however, it may also be used to build defense enzymes, which can be important for plant survival [12], [14]. Our model implicitly incorporated this type of investment in the storage nitrogen; however, for a better understanding of the tradeoffs between plant growth and persistence, it is important that future field and modeling study quantify this type of nitrogen investment.

Third, the model assumes that nitrogen allocation to light capture, electron transport, carboxylation, and respiration are always optimized to maximize photosynthesis and that growth nitrogen is balanced with storage nitrogen as determined by the nitrogen storage duration parameter. In the real world, it may take plants time to reach this optimization or balance depending on prevailing environmental conditions. In our test cases, the treatment time varied from months for an herbaceous plant to years for trees to show nitrogen allocation acclimation; however, there is a limited amount of data available to specifically determine acclimation times.

Finally, the model assumes complete acclimation to changing environmental conditions. In the real world, plants may have limited genetic acclimation capability and might not be able to reach an optimal balance of nitrogen pools. For example, in test case 3 where a radiation reduction experiment (reduced from 450 to 50 *µmol* photon/*m* ^2^/*s*) was conducted, the predicted “optimal” *V_c,max_* under the low radiation exposure is much lower than the measured *V_c,max_* (Figure S6 a), suggesting a potential acclimation limitation. Interestingly, the plants appear to acclimate to the temperature reduction (Figure S5 a). This is presumably because our model predicts a larger change in nitrogen allocation (e.g., nitrogen allocated to carboxylation) for radiation reduction compared to that for the decreased temperature (Figure S5 c–e and Figure S6 c–e). More extensive datasets are required to get a better estimation of plant acclimation ability for models that simulate individual species; however, for global dynamic vegetation model that targets different plant functional types, this could be less an issue because the large variety of species within functional types provide greater acclimation potential as a community than any single species.

## Supporting Information

Text S1 **Description of the nitrogen allocation model.**
(DOCX)

Text S2 **Nitrogen use efficiencies.**
(DOCX)

Text S3 **Photosynthesis model.**
(DOCX)

Text S4 **Fitting nitrogen allocation model to data.**
(DOCX)

Text S5 **Temperature dependence of key model parameters.**
(DOCX)

Text S6 ***V*** **_c,max_ and** ***J_max_*** **standardization.**
(DOCX)

Figure S1 **Sensitivity analysis of the relationship between** ***V_c,max25_*** **and leaf nitrogen to changes in proportion of respiratory nitrogen allocated to leaf (** ***f_r_*** **).** *f_r_* increases from 0.4 to 0.6. The nitrogen storage duration is set to be 65 days. Other parameters are from test case 1 in Table 1.
(TIF)

Figure S2 **Sensitivity analysis of the relationship between** ***V_c,max25_*** **and leaf nitrogen to changes in proportion of storage nitrogen allocated to leaf (** ***f_s_*** **).** *f_s_* increases from 0.3 to 0.8. The nitrogen storage duration is set to be 65 days. Other parameters are from test case 1 in Table 1.
(TIF)

Figure S3 **Sensitivity analysis of the relationship between** ***V_c,max25_*** **and leaf nitrogen to changes in nitrogen storage duration (** ***D_ns_*** **).** *D_ns_* increases from 5 to 65. The proportion of storage nitrogen allocated to leaf (*f_s_*) is set to be 0.8. Other parameters are from test case 1 in Table 1.
(TIF)

Figure S4 **CO_2_ fertilization effects on leaf nitrogen allocation for test case one.** In panel (a), closed and open circles represent observed *V_c,max_* scaled to 25°C (i.e.,*V_c,max25_*)for loblolly pine (*Pinus taeda*) with ambient (370 *ppm*) and elevated CO_2_ concentration (570 *ppm*), respectively. Solid lines are estimates of *V_c,max25_* by the nitrogen allocation model tuned to ambient CO_2_ data, while dashed lines are predictions of *V_c,max25_* by the tuned nitrogen allocation model using elevated CO_2_ concentration. Panels (b)–(f) show the fitted (solid lines) and predicted (dashed lines) proportion of leaf nitrogen allocated to storage, carboxylation, light capture, electron transport, and respiration, respectively. See Table 1 for main model inputs and Table 2 for fitted parameter values.
(TIF)

Figure S5 **Growing temperature effects on leaf nitrogen allocation for test case three.** In panel (a), open and filled circles indicate observed *V_c,max_* scaled to 25°C (i.e.,*V_c,max25_*) for a Japanese plantain (*Plantago asiatica*) growing at temperatures of 15°C and 30°C, respectively, both of which are scaled to the reference temperature of 25°C. Plants in both treatments were growing at a relatively high radiation exposure (450 *µmol* photon/*m* ^2^/*s* for 4 hours and 50 *µmol* photon/*m* ^2^/*s* for 10 hours). Solid lines are estimates of *V_c,max25_* by the nitrogen allocation model tuned to data at the high growing temperature (30°C), while dashed lines are predictions of *V_c,max25_* by the tuned nitrogen allocation model using the low growing temperature (15°C). Panels (b)–(f) show the fitted (solid lines) and predicted (dashed lines) proportion of leaf nitrogen allocated to storage, carboxylation, light capture, electron transport, and respiration, respectively. See Table 1 for main model inputs and Table 2 for fitted parameter values.
(TIF)

Figure S6 **Radiation effects on leaf nitrogen allocation for test case three.** In panel (a), open and closed circles represent observed *V_c,max_* scaled to 25°C (i.e.,*V_c,max25_*) for a Japanese plantain (*Plantago asiatica*) growing at a low (50 *µmol* photon/*m* ^2^/*s*) and high radiation (450 *µmol* photon/*m* ^2^/*s*) exposure, respectively. Plants in both treatments were growing at a relatively low temperature (15°C). Solid lines are predictions of *V_c,max25_* by the nitrogen allocation model fitted to data at a high growing temperature (30°C) and a high level of radiation(450 *µmol* photon/*m^2^/s*) (see filled circles in Figure S6 a). Dashed lines are predictions of *V_c,max25_* by the fitted nitrogen allocation model using a radiation level of 300 *µmol* photon /*m* ^2^/*s*, assume a partial acclimation. Dotted grey lines are predictions of *V_c,max25_* by the fitted nitrogen allocation model using a radiation level of 50 *µmol*/*m* ^2^/*s*, assuming a complete acclimation. Panels (b)–(f) show the fitted (solid lines) and predicted (dashed lines) proportion of leaf nitrogen allocated to storage, carboxylation, light capture, electron transport, and respiration, respectively. See Table 1 for main model inputs and Table 2 for fitted parameter values.
(TIF)

Table S1 **Main model parameters.**
(DOCX)

## References

[1] LeBauer DS, Treseder KK (2008). Nitrogen limitation of net primary productivity in terrestrial ecosystems is globally distributed.. Ecology.

[2] Chapin FS, Shaver GR (1985). Individualistic growth response of tundra plant species to environmental manipulations in the field.. Ecology.

[3] Shaver GR, Bret-Harte SM, Jones MH, Johnstone J, Gough L (2001). Species composition interacts with fertilizer to control long-term change in tundra productivity.. Ecology.

[4] Aber JD, Ollinger SV, Driscoll CT (1997). Modeling nitrogen saturation in forest ecosystems in response to land use and atmospheric deposition.. Ecological Modelling.

[5] Thornton PE, Law BE, Gholz HL, Clark KL, Falge E (2002). Modeling and measuring the effects of disturbance history and climate on carbon and water budgets in evergreen needleleaf forests.. Agricultural and Forest Meteorology.

[6] Reich PB, Walters MB, Kloeppel BD, Ellsworth DS (1995). Different photosynthesis-nitrogen relations in deciduous hardwood and evergreen coniferous tree species.. Oecologia.

[7] Friend AD (1991). Use of a model of photosynthesis and leaf microenvironment to predict optimal stomatal conductance and leaf nitrogen partitioning.. Plant Cell and Environment.

[8] Ripullone F, Grassi G, Lauteri M, Borghetti M (2003). Photosynthesis–nitrogen relationships: interpretation of different patterns between Pseudotsuga menziesii and Populus×euroamericana in a mini-stand experiment.. Tree Physiology.

[9] Tang J, Zhuang Q (2009). A global sensitivity analysis and Bayesian inference framework for improving the parameter estimation and prediction of a process-based Terrestrial Ecosystem Model.. Journal of Geophysical Research.

[10] Bonan GB, Lawrence PJ, Oleson KW, Levis S, Jung M (2011). Improving canopy processes in the Community Land Model version 4 (CLM4) using global flux fields empirically inferred from FLUXNET data.. Journal of Geophysical Research-Biogeosciences.

[11] Marschner H (1995). Mineral nutrition of higher plants.

[12] Herms DA, Mattson WJ (1992). The dilemma of plants: to grow or defend.. The Quarterly Review of Biology.

[13] Verkroost AWM, Wassen MJ (2005). A simple model for nitrogen-limited plant growth and nitrogen allocation.. Annals of Botany.

[14] Chapin FS, Schulze E, Mooney HA (1990). The ecology and economics of storage in plants.. Annual Review of Ecology and Systematics.

[15] Evans JR, Poorter H (2001). Photosynthetic acclimation of plants to growth irradiance: the relative importance of specific leaf area and nitrogen partitioning in maximizing carbon gain.. Plant, Cell & Environment.

[16] Evans JR (1989). Photosynthesis and nitrogen relationships in leaves of C_3_ and C_4_ plants.. Oecologia.

[17] Makino A, Osmond B (1991). Effects of nitrogen nutrition on nitrogen partitioning between chloroplasts and mitochondria in pea and wheat.. Plant Physiology.

[18] Bendall DS (1958). Cytochromes and Some Respiratory Enzymes in Mitochondria from the Spadix of Arum-Maculatum.. Biochemical Journal.

[19] Bloom AJ, Chapin FS, Mooney HA (1985). Resource limitation in plants - an economic analogy.. Annual Review of Ecology and Systematics.

[20] Millard P (1988). The accumulation and storage of nitrogen by herbaceous plants.. Plant, Cell & Environment.

[21] Kleijn D, Treier UA, Müller-Schärer H (2005). The Importance of Nitrogen and Carbohydrate Storage for Plant Growth of the Alpine Herb Veratrum album.. New Phytologist.

[22] Hikosaka K, Terashima I (1995). A model of the acclimation of photosynthesis in the leaves of C3 plants to sun and shade with respect to nitrogen use.. Plant, Cell & Environment.

[23] Niinemets U, Tenhunen JD (1997). A model separating leaf structural and physiological effects on carbon gain along light gradients for the shade-tolerant species Acer saccharum.. Plant Cell and Environment.

[24] White MA, Thornton PE, Running SW, Nemani RR (2000). Parameterization and sensitivity analysis of the BIOME–BGC terrestrial ecosystem model: net primary production controls.. Earth Interactions.

[25] Dewar RC, Franklin O, Mäkelä A, McMurtrie RE, Valentine HT (2009). Optimal Function Explains Forest Responses to Global Change.. Bioscience.

[26] Oaks A, Stulen I, Jones K, Winspear M, Misra S (1980). Enzymes of nitrogen assimilation in maize roots.. Planta.

[27] Niinemets U (1997). Role of foliar nitrogen in light harvesting and shade tolerance of four temperate deciduous woody species.. Functional Ecology.

[28] Long SP, Ainsworth EA, Rogers A, Ort DR (2004). Rising atmospheric carbon dioxide: plants face the future.. Annual Review of Plant Biology.

[29] Seiwa K (2007). Trade-offs Between Seedling Growth and Survival in Deciduous Broadleaved Trees in a Temperate Forest.. Annals of Botany.

[30] Crous KY, Walters MB, Ellsworth DS (2008). Elevated CO_2_ concentration affects leaf photosynthesis-nitrogen relationships in Pinus taeda over nine years in FACE.. Tree Physiology.

[31] Niinemets Ü, Kull O, Tenhunen JD (1998). An analysis of light effects on foliar morphology, physiology, and light interception in temperate deciduous woody species of contrasting shade tolerance.. Tree Physiology.

[32] Hikosaka K (2005). Nitrogen partitioning in the photosynthetic apparatus of Plantago asiatica leaves grown under different temperature and light conditions: Similarities and differences between temperature and light acclimation.. Plant and Cell Physiology.

[33] Metropolis N, Rosenbluth AW, Rosenbluth MN, Teller AH, Teller E (1953). Equation of state calculations by fast computing machines.. Journal of Chemical Physics.

[34] Hastings WK (1970). Monte Carlo sampling methods using Markov chains and their applications.. Biometrika.

[35] Collatz GJ, Ball JT, Grivet C, Berry JA (1991). Physiological and environmental regulation of stomatal conductance, photosynthesis and transpiration: a model that includes a laminar boundary layer.. Agricultural and Forest Meteorology.

[36] Holaday AS, Martindale W, Alred R, Brooks AL, Leegood RC (1992). Changes in activities of enzymes of carbon metabolism in leaves during exposure of plants to low-temperature.. Plant Physiology.

[37] Ehleringer J, Björkman O (1977). Quantum Yields for CO2 Uptake in C3 and C4 Plants: Dependence on Temperature, CO2, and O2 Concentration.. Plant Physiology.

[38] Leegood RC, Edwards GE, Baker NR (2004). Carbon Metabolism and Photorespiration: Temperature Dependence in Relation to Other Environmental Factors..

[39] Makino A, Nakano H, Mae T (1994). Effects of growth temperature on the responses of ribulose-1,5-biphosphate carboxylase, electron transport components, and sucrose synthesis enzymes to leaf nitrogen in rice, and their relationships to photosynthesis.. Plant Physiology.

[40] Haldimann P (1998). Low growth temperature-induced changes to pigment composition and photosynthesis in Zea mays genotypes differing in chilling sensitivity.. Plant, Cell & Environment.

[41] Farquhar GD, von Caemmerer S, Berry JA (1980). A biochemical model of photosynthetic CO_2_ assimilation in leaves of C_3_ species.. Planta.

[42] Reich P, Oleksyn J, Wright I (2009). Leaf phosphorus influences the photosynthesis–nitrogen relation: a cross-biome analysis of 314 species.. Oecologia.

[43] Medlyn BE, Badeck FW, De Pury DGG, Barton CVM, Broadmeadow M (1999). Effects of elevated [CO_2_] on photosynthesis in European forest species: a meta-analysis of model parameters.. Plant, Cell & Environment.

[44] Ainsworth EA, Long SP (2005). What have we learned from 15 Years of Free-Air CO_2_ Enrichment (FACE)? A meta-analytic review of the responses of photosynthesis, canopy properties and plant production to rising CO_2_.. New Phytologist.

[45] Norby RJ, DeLucia EH, Gielen B, Calfapietra C, Giardina CP (2005). Forest response to elevated CO_2_ is conserved across a broad range of productivity.. Proceedings of the National Academy of Sciences of the United States of America.

[46] Hikosaka K, Terashima I (1996). Nitrogen partitioning among photosynthetic components and its consequence in sun and shade plants.. Functional Ecology.

[47] Niinemets U, Kull O, Tenhunen JD (1998). An analysis of light effects on foliar morphology, physiology, and light interception in temperate deciduous woody species of contrasting shade tolerance.. Tree Physiology.

[48] McMurtrie RE, Dewar RC (2011). Leaf-trait variation explained by the hypothesis that plants maximize their canopy carbon export over the lifespan of leaves.. Tree Physiology.

[49] Fisher JB, Sitch S, Malhi Y, Fisher RA, Huntingford C (2010). Carbon cost of plant nitrogen acquisition: A mechanistic, globally applicable model of plant nitrogen uptake, retranslocation, and fixation.. Global Biogeochem Cycles.

[50] Nordin A, Nasholm T (1997). Nitrogen storage forms in nine boreal understorey plant species.. Oecologia.

[51] Warren CR, Dreyer E, Adams MA (2003). Photosynthesis-Rubisco relationships in foliage of Pinus sylvestris in response to nitrogen supply and the proposed role of Rubisco and amino acids as nitrogen stores.. Trees-Structure and Function.

[52] Seemann JR, Sharkey TD, Wang J, Osmond CB (1987). Environmental Effects on Photosynthesis, Nitrogen-Use Efficiency, and Metabolite Pools in Leaves of Sun and Shade Plants.. Plant Physiology.

[53] Perez R, Ineichen P, Moore K, Kmiecik M, Chain C (2002). A new operational model for satellite-derived irradiances: Description and validation.. Solar Energy.

[54] Kalnay E, Kanamitsu M, Kistler R, Collins W, Deaven D (1996). The NCEP/NCAR 40-year reanalysis project.. Bulletin of the American Meteorological Society.

[55] Thornton PE, Running SW, White MA (1997). Generating surfaces of daily meteorological variables over large regions of complex terrain.. Journal of Hydrology.

[56] Rogers A, Ellsworth DS (2002). Photosynthetic acclimation of Pinus taeda (loblolly pine) to long-term growth in elevated pCO2 (FACE).. Plant, Cell & Environment.

[57] Niinemets Ü, Kull O (1998). Stoichiometry of foliar carbon constituents varies along light gradients in temperate woody canopies: implications for foliage morphological plasticity.. Tree Physiology.

[58] Kobayashi T, Okamoto K, Hori Y (2001). Variations in size structure, growth and reproduction in Japanese plantain (Plantago asiatica L.) between exposed and shaded populations.. Plant Species Biology.

[59] McCarthy HR, Oren R, Johnsen KH, Gallet-Budynek A, Pritchard SG (2010). Re-assessment of plant carbon dynamics at the Duke free-air CO(2) enrichment site: interactions of atmospheric [CO(2)] with nitrogen and water availability over stand development.. New Phytologist.

[60] Poorter H, Remkes C, Lambers H (1990). Carbon and Nitrogen Economy of 24 Wild-Species Differing in Relative Growth-Rate.. Plant Physiology.

