# Supplementary material for: Toward a Mechanistic Modeling of Nitrogen Limitation on Vegetation Dynamics
Source: PLoS One. 2012 May 23;7(5):e37914. doi: 10.1371/journal.pone.0037914 (PMC3359379; doi:10.1371/journal.pone.0037914)
Supplement: Text S1 — Description of the nitrogen allocation model. (DOCX) [file pone.0037914.s001.docx]

**Text S1: Description of the nitrogen allocation model**

1. **Growth and storage nitrogen allocation**

The proportion of storage nitrogen in the functional nitrogen pool is determined by a nitrogen storage duration parameter$D_{ns}$, the net carbon assimilation rate ($A_{n}$) and the target sink tissue nitrogen requirement ($NR{}_{sink}$, *g* N/*g* biomass) as follows,

$D_{ns}A_{n}NR{}_{sink}=(1-PN_{g})FNA_{a} .$ (S1.1)

The left side of the equations is the demand of storage nitrogen, while the right side of the equation is the supply of storage nitrogen. See Table S1 for definitions of main model parameters. In this equation, the net carbon assimilation rate ($A_{n}$) is calculated by subtracting respiration from gross carbon assimilate rate,

$A_{n}=C_{v}[(1-C_{gr})NUE_{p}PN_{p}PN_{g}FNA_{a}-R_{m})]$ (S1.2)

where the gross carbon assimilate rate (i.e., $NUE_{p}PN_{p}PN_{g}FNA_{a}$) equals the size of the photosynthetic nitrogen pool ($PN_{p}PN_{g}FNA_{a}$, see Figure 1 for a better understanding) multiplied by the photosynthetic nitrogen use efficiency ($NUE_{p}$, *µmol* CO_2_/*g* photosynthetic N/day, see Text S2 for details). Net carbon assimilation is the gross carbon assimilate rate minus the growth respiration fraction ($C_{gr}$) and the maintenance respiration (*R*_m_, *µmol* CO_2_/m^2^/day). We assume that 25% of gross carbon assimilation is used for growth respiration [1,2] and the maintenance respiration is dependent on functional nitrogen content as follows [3],

(S1.3)

where $MR_{b}$ is the maintenance respiration demand per gram of nitrogen (*µmol* CO_2_/*g* functional nitrogen/day). We specifically not include the structural nitrogen for the calculation of respiration in view that the structural nitrogen is not functionally active and it may require little energy to maintain the structural nitrogen. See Text S2 for details of estimation of$MR_{b}$.

The $NR{}_{sink}$ is the nitrogen requirement per gram of new tissue biomass and is calculated as the sum of functional nitrogen requirement and structural nitrogen requirement. It can be estimated as follows,

$NR{}_{sink}=(\eta_{l}\cdot FNA_{m,ref}+SNC_{m})$ (S1.4)

where $FNA_{m,ref}$ (*g* plant functional N/*g* leaf) is amount of plant functional nitrogen required to support the growth and maintenance of one gram of new leaf. This required plant functional nitrogen includes the functional nitrogen in leaves as well as the functional nitrogen in roots and sapwood, which is used to acquire water and nutrient for photosynthesis and to provide nitrogen for new tissue synthesis using the photosynthetic products. $SNC_{m}$ (g structural N/g plant biomass) is the structural nitrogen content and is set to be 0.001 based on C:N ratio data from dead wood [4]. $FNA_{m,ref}$may be able to be estimated from field data that have a complete nitrogen budget measurement; however, it would be challenging to get reliable data because it would be difficult to measure the underground components. Thus, in this paper, we propose to estimate$FNA_{m,ref}$ from the measured mean leaf nitrogen content (*g* N/*g* leaf biomass,$MLNC_{m}$) as follows

$FNA_{m,ref}=k MLNC_{m}$, (S1.5)

where the coefficient *k* is the ratio of total plant functional nitrogen to the amount of total nitrogen allocated to leaf. Because we will tune the nitrogen storage duration parameter (*D_ns_*) to fit our model to the *V_c,max_* data, an under-estimation or over-estimation of $k$will be compensated by larger or smaller values of *D_ns_*. To improve the parameter identifiability in model fitting, we empirically fix the value of *k* at 1.1, in view that the majority of functional nitrogen is allocated to leaf. Replacing eqs. () and () into eq. (), we have

(S1.6)

We solve eq. () to derive given the values of $PN_{p}$and $FNA_{a}$.

1. **Photosynthetic and respiratory nitrogen allocation**

The growth nitrogen is partitioned into photosynthetic and respiratory organelles. We assume that 25% of photosynthesis production is used for growth respiration [1] and maintenance respiration is dependent on functional nitrogen content (see eq. ()). To maximize the carbon gain given a certain amount of growth nitrogen, we equalize the nitrogen allocated to respiratory organelles to the rate of respiration implied by the growth and maintenance terms,

$\frac{C_{gr}NUE_{p}PN_{p}PN_{g}FNA_{a}+R_{m}}{NUE_{r}}=(1-PN_{p})PN_{g}FNA_{a}$ (S1.7)

where the numerator on the leaf side of equation specifies the carbon used in growth respiration and maintenance respiration. This is divided by the nitrogen use efficiency of respiratory enzymes ($NUE_{r}$, *µmol* CO_2_/*g* respiratory N/day, see Text S2 for details) to give the nitrogen demand for respiration. The right hand side represents nitrogen supply for respiration as opposed to photosynthesis.

1. **Light captures and electron transport nitrogen allocation**

The actual electron transportation rate is dependent on photosynthetic active radiation, light harvesting rate, and maximum electron transportation rate [5,6]. The light harvesting rate ($J_{l}$, *µ*mol electron/*m*^2^/*s*) can be estimated based on photosynthetic active radiation ($PAR$; µmol photon/*m*^2^/*s*) and light absorption efficiency ($\alpha$) as follows [6]

$J_{l}=0.292\alpha PAR$ (S1.8)

with

$\alpha=\frac{[Chl]}{0.076+[Chl]}$ (S1.9)

where $[Chl]$ is the chlorophyll content (*mmol* Chl/m^2^) and the coefficient 0.292 converts $\alpha$; electron/photon. The chlorophyll content can be determined by the proportion of nitrogen allocated for light absorption as follows,

$[Chl]=1.78PN_{chl}PN_{l}PNC_{a}$ (S1.10)

where $PN_{chl}$ is the proportion of nitrogen allocated for light absorption within the light-harvesting nitrogen pool (See Figure 1 for details). The coefficient 1.78 is the nitrogen binding coefficient for chlorophyll (*mmol* Chl/*g* N) [7].

To maximize the carbon gain given a certain amount of light harvesting nitrogen, we equalize the daytime mean light harvesting rate with the maximum electron transportation rate ($J_{\max}$). Namely,

$J_{\max}=J_{l}$ (S1.11)

$J_{\max}$depends on the amount of nitrogen allocated to electron transport ($1-PN_{chl}$) as opposed to light capture. Specifically,

$J_{\max}=NUE_{J}(1-PN_{chl})PN_{l}PNC_{a}$ (S1.12)

where $PN_{l}PNC_{a}$ specifies the light-harvesting nitrogen content (*g* N/*m*^2^ leaf) within the photosynthetic nitrogen pool.$NUE_{J}$ is the nitrogen use efficiency for electron transport (*µmol* electron/*g* N/*s*). See Text S2 for details of $NUE_{J}$estimation. Replacing eqs. (S), (), (), and () into eq.(S), we have

(S1.13)

We solve eq. () to estimate $PN_{chl}$ given values of$PN_{l}$ and $PNC_{a}$.

The actual electron transportation rate is estimated using the Smith's equation as follows [5,6]

$J=J_{\max}\frac{0.292\alpha PAR}{\sqrt{J_{\max}^{2}+(0.292\alpha PAR)^{2}}}$. (S1.14)

1. **Light harvesting and carboxylation nitrogen allocation**

Finally, based on the electron transport rates calculated above, photosynthetic nitrogen is allocated between light-harvesting and carboxylation by equalizing the Rubisco-limited carboxylation rate (*W_c_*) and electron-transport-limited carboxylation rate (*W_j_*). Following the Farquhar model [8], Rubisco-limited carboxylation rate, $W_{c}$, is estimated as follows,

$W_{c}=R_{c}V_{c, max}$ (S1.15)

where $V_{cmax}$is the maximum rate of carboxylation (*µmol* CO_2_/*m*^2^/*s*) and $R_{c}$ is the CO_2_ concentration adjustment factor. See Text S3 for details of $R_{c}$ calculation. The value of$V_{cmax}$ is determined by the nitrogen allocated to carboxylation as follows,

$V_{c, max}=NUE_{c}(1-PN_{l})PNC_{a}$ , (S1.16)

where $(1-PN_{l})PNC_{a}$ specifies the carboxylation nitrogen content (*g* N/*m*^2^ leaf) within the photosynthetic nitrogen pool. $NUE_{c}$ is the nitrogen use efficiency for maximum rate of carboxylation (*µmol* CO_2_ /*g* N/*s*). See Text S2 for details of $NUE_{c}$ calculation.

The electron-transport-limited carboxylation rate can be estimated based on the potential electron transport rate [9],

$W_{j}=R_{j}J$, (S1.17)

where $R_{j}$ is the CO_2_ concentration adjustment factor. See Text S3 for details of $R_{j}$ calculation.

To maximize the carbon gain given a certain amount of photosynthetic nitrogen, we equalize $W_{c}$and $W_{j}$. This leads to the following equation,

$\frac{5.2NUE_{J}(1-PN_{chl})PN_{chl}PN_{l}R_{j}PAR}{\sqrt{[NUE_{J}(1-PN_{chl})(0.076+17.8PN_{chl}PN_{l}PNC_{a})]^{2}+(5.2PN_{chl}PAR)^{2}}}=R_{c}NUE_{c}(1-PN_{l})$. (S1.18)

The nitrogen allocation for light harvesting is thus estimated by solving for $PN_{l}$in the above equation given values of $PN_{chl}$and $PNC_{a}$.

**Literature**

1. Baker DN, Hesketh JD, Duncan WG (1971) Simulation of growth and yield in cotton.I. gross photosynthesis, respiration, and growth. Crop Science 12: 431-435.

2. Williams K, Percival F, Merino J, Mooney HA (1987) Estimation of tissue construction cost from heat of combustion and organic nitrogen content. Plant, Cell & Environment 10: 725-734.

3. Ryan MG (1991) A simple method for estimating gross carbon budgets for vegetation in forest ecosystems. Tree Physiology 9: 255-266.

4. White MA, Thornton PE, Running SW, Nemani RR (2000) Parameterization and sensitivity analysis of the BIOME–BGC terrestrial ecosystem model: net primary production controls. Earth Interactions 4: 1-85.

5. Tenhunen JD, Weber JA, Yocum CS, Gates DM (1976) Development of a Photosynthesis Model with an Emphasis on Ecological Applications. II. Analysis of a Data Set Describing the $P_{M}$ Surface. Oecologia 26: 101-119.

6. Niinemets U, Tenhunen JD (1997) A model separating leaf structural and physiological effects on carbon gain along light gradients for the shade-tolerant species Acer saccharum. Plant Cell and Environment 20: 845-866.

7. Evans JR (1989) Photosynthesis and nitrogen relationships in leaves of C_3_ and C_4_ plants. Oecologia 78: 9-19.

8. Farquhar GD, von Caemmerer S, Berry JA (1980) A biochemical model of photosynthetic CO_2_ assimilation in leaves of C_3_ species. Planta 149: 78-90.

9. Long SP (1991) Modification of the response of photosynthetic productivity to rising temperature by atmospheric CO_2_ concentrations - has its importance been underestimated. Plant Cell and Environment 14: 729-739.
