# Supplementary material for: Toward a Mechanistic Modeling of Nitrogen Limitation on Vegetation Dynamics
Source: PLoS One. 2012 May 23;7(5):e37914. doi: 10.1371/journal.pone.0037914 (PMC3359379; doi:10.1371/journal.pone.0037914)
Supplement: Text S2 — Nitrogen use efficiencies. (DOCX) [file pone.0037914.s002.docx]

**Text S2: Nitrogen use efficiencies**

The carboxylation nitrogen use efficiency (*µmol* CO_2_/ *g* N/*s*),$NUE_{c}$, is estimated as follows,

$NUE_{c}=0.8\times33.3\times6.25\times f_{V_{c,max}}\text{(}T\text{)}$ (S2.1)

where the constant 33.3 is the specific RUBISCO activity (*µmol* CO_2_/*g* Rubisco/*s*) measured at 25 ^o^C [1] and the constant 6.25 is the nitrogen binding factor for Rubisco (*g* Rubisco /*g* N) . Although Rubisco is the main carboxylation enzyme, there are many other Calvin Cycle enzymes involved in carboxylation [2]. Currently, few data are available in terms of the ratio of nitrogen invested in Rubisco to other Calvin Cycle enzymes. In this study we use an empirical value of 0.8 [2]. $f_{V_{c,max}}\text{(}T\text{)}$is the function specifying the temperature dependence of$V_{cmax}$. See Text S3 for details of $f_{V_{c,max}}\text{(}T\text{)}$estimation.

The nitrogen use efficiency for maximum electron transport (*µmol* electron/*g* N/*s*), $NUE_{J}$, is estimated based on a characteristic protein cytochrome *f* [3],

$NUE_{J}$=$8.06\times156\times f_{Jmax}(T)$ (S2.2)

where the coefficient 156 is the maximum electron transport rate for cytochrome *f* at 25^o^C(*µmol* electron*/µmol* cytochrome *f*); 8.06 is the nitrogen binding coefficient for cytochrome *f* (*µmol* cytochrome *f /g N* in bioenergetics). $f_{Jmax}(T)$is a function specifies the dependence of $J_{\max}$on temperature ( see Text S3). Notice that, because $J_{\max}$is assumed to be temperature-dependant and light absorption is not temperature-dependant, we specifically distinguish nitrogen allocation for light absorption and for electron transport in photosystem I, II and light-harvesting complexes. See Text S5 for details of $f_{Jmax}(T)$estimation.

The photosynthetic nitrogen use efficiency (*µmol* CO_2_/*g* N/day),$NUE_{p}$, can be estimated using the Farquhar photosynthesis model as follows,

$NUE_{p}=\frac{\sum_{i=1}^{n_{dstep}} \frac{D_{day}}{n_{dstep}}Min (W_{c},W_{j})}{PNC_{a}}$, (S2.3)

where $W_{j}$is electron-limited rate and $W_{c}$ is Rubiso-limited rate as follows. $n_{dstep}$is the number of daytime step to calculate daily photosynthesis rate. $D_{day}$is the duration of day time (seconds). See text S4 for details of $W_{c}$ and $W_{j}$ calculations. Notice that $W_{j}$ may change with radiation variation within a day.

The nitrogen use efficiency of enzymes for respiration, $NUE_{r}$, is assumed to be temperature-dependant. Specifically, it is calculated as follows,

$NUE_{r}=33.69 [D_{day}f_{r}(T_{day})+D_{night}f_{r}(T_{night})]$ (S2.4)

where 33.69 is the specific nitrogen use efficiency for respiration at 25^o^C (*µmol* CO_2_ /*g* N/*s*) [5] and $f_{r}(T)$specifies the dependence of $NUE_{r}$on temperature (See Text S3 for details). $D_{day}$ and $D_{night}$ is the daytime and nighttime length in seconds.

The maintenance respiration is modeled to be dependent on the amount of functional nitrogen in plant (see eq. (2) in main text). The maintenance respiration demand per gram of nitrogen, $MR_{b}$, is estimated during the day as follows,

$MR_{b}=0.21[D_{day}f_{r}(T_{day})+D_{night}f_{r}(T_{night})]$ (S2.5)

where the coefficient 0.21 is the nitrogen demand at 25^o^C (*µmol CO_2_/g* functional nitrogen */s*) [6].

**Literatures:**

1. Rintamäki E, Keys AJ, Parry MAJ (1988) Comparison of the specific activity of ribulose-1,5-bis-phosphate carboxylase-oxygenase from some C3 and C4 plants. Physiologia Plantarum 74: 326-331.

2. Hikosaka K, Terashima I (1996) Nitrogen partitioning among photosynthetic components and its consequence in sun and shade plants. Functional Ecology 10: 335-343.

3. Niinemets U, Tenhunen JD (1997) A model separating leaf structural and physiological effects on carbon gain along light gradients for the shade-tolerant species Acer saccharum. Plant Cell and Environment 20: 845-866.

4. Evans JR (1989) Photosynthesis and nitrogen relationships in leaves of C_3_ and C_4_ plants. Oecologia 78: 9-19.

5. Makino A, Osmond B (1991) Effects of nitrogen nutrition on nitrogen partitioning between chloroplasts and mitochondria in pea and wheat. Plant Physiology 96: 355-362.

6. Ryan MG (1991) A simple method for estimating gross carbon budgets for vegetation in forest ecosystems. Tree Physiology 9: 255-266.
