# Supplementary material for: Toward a Mechanistic Modeling of Nitrogen Limitation on Vegetation Dynamics
Source: PLoS One. 2012 May 23;7(5):e37914. doi: 10.1371/journal.pone.0037914 (PMC3359379; doi:10.1371/journal.pone.0037914)
Supplement: Text S3 — Photosynthesis model. (DOCX) [file pone.0037914.s003.docx]

**Text S4: Photosynthesis model**

The photosynthesis rate can be limited by carboxylation enzyme (mainly Rubisco) or electron transport. Following the Farquhar model [1], Rubisco-limited carboxylation rate is estimated as follows,

$W_{c}=R_{c}V_{c,max}$ (S3.1)

where $V_{c,max}$is the maximum rate of carboxylation (*µmol* CO_2_/*m*^2^/*s*). $R_{c}$ is the CO_2_ concentration adjustment factor ,

$R_{c}=\frac{\text{max(}C_{i}-C_{p}\text{,0)}}{C_{i}\text{+}K_{c}\text{(1.0+[O}_{2}\text{]/}K_{o}\text{)}}$, (S3.2)

where $C_{i}$is the leaf internal CO_2_ concentration (Pa) and $C_{p}$the CO_2_ compensation point (Pa). $K_{c}$and $K_{o}$ are the Michaelis constant for CO_2_ and the competitive inhibition constant for O_2_ in Rubisco reactions, respectively. $C_{p}$ is calculated as a function of $K_{c}$,$K_{o}$ and O_2_ concentration in the water (Pa) as follows [2],

$C_{p}=0.105 [O_{2}]K_{c}/K_{o}$. (S3.3)

Refer to Collatz et al [3] for values and temperature dependence of $K_{c}$and $K_{o}$. The internal leaf CO_2_ concentration is estimated based on air CO_2_ concentration and stomata conductance [3]. The empirical equation of Ball and Berry [4] , which links stomata conductance with photosynthesis, air humidity and air CO_2_ concentration , is used to estimate the stomata conductance [3].

The electron-transport-limited carboxylation rate can be estimated based on the potential electron transport rate as follows [2],

$W_{j}=R_{j}J$, (S3.4)

where $R_{j}$ is the CO_2_ concentration adjustment factor

$R_{j}=\frac{\text{max(}C_{i}-C_{p}\text{,0)}}{4C_{i}\text{+8}C_{p}}$ . (S3.5)

**Literature**

1. Farquhar GD, Caemmerer S, Berry JA (1980) A biochemical model of photosynthetic CO_2_ assimilation in leaves of C_3_ species. Planta 149: 78-90.

2. Long SP (1991) Modification of the response of photosynthetic productivity to rising temperature by atmospheric CO_2_ concentrations - has its importance been underestimated. Plant Cell and Environment 14: 729-739.

3. Collatz GJ, Ball JT, Grivet C, Berry JA (1991) Physiological and environmental regulation of stomatal conductance, photosynthesis and transpiration: a model that includes a laminar boundary layer. Agricultural and Forest Meteorology 54: 107-136.

4. Ball JT, Woodrow IE, Berry JA (1987) A model predicting stomatal conductance and its contribution to the control of photosynthesis under different environmental conditions. In: Biggins J, editor. Progress in Photosynthesis Research. Netherlands: Martinus Nijhoff Publishers. pp. 221–224.
