# Supplementary material for: Toward a Mechanistic Modeling of Nitrogen Limitation on Vegetation Dynamics
Source: PLoS One. 2012 May 23;7(5):e37914. doi: 10.1371/journal.pone.0037914 (PMC3359379; doi:10.1371/journal.pone.0037914)
Supplement: Text S4 — Fitting nitrogen allocation model to data. (DOCX) [file pone.0037914.s004.docx]

**Text S4: Fitting nitrogen allocation model to data**

For a specific nitrogen storage duration parameter ($D_{ns}$), we can iteratively solve eqs. (S1.6), (S1.7), (S1.13), and (S1.18) in Text S1 to estimate the hierarchical nitrogen allocation coefficients for growth ($PN_{g}$), photosynthesis ($PN_{p}$), light absorption ($PN_{chl}$) and light harvesting ($PN_{l}$). Using the hierarchical nitrogen allocation coefficients, we are able to estimate the proportions of storage nitrogen ($PNF_{s}$), respiratory nitrogen ($PNF_{r}$), carboxylation nitrogen ($PNF_{c}$), light capture nitrogen ($PNF_{l}$) and electron transport nitrogen ($PNF_{e}$) within the functional nitrogen pool as follows (see Fig. 1 for a better understanding),

$\begin{matrix} PNF_{s}=1-PN_{g} \\ PNF_{r}=(1-PN_{p})PN_{g} \\ PNF_{c}=(1-PN_{l})(1-PN_{p})PN_{g} \\ PNF_{l}=PN_{chl}(1-PN_{l})(1-PN_{p})PN_{g} \\ PNF_{e}=(1-PN_{chl})(1-PN_{l})(1-PN_{p})PN_{g} \end{matrix}$. (S4.1)

Many studies reported the relationship between *V_c,max_* and leaf nitrogen content. To fit our model to the observed values of *V_c,max_* at different levels of leaf nitrogen content, we need to first estimate the leaf nitrogen content based on the functional nitrogen content. Specifically, leaf-area-based nitrogen content ($LNC_{a}$, *g* N/*g* leaf biomass) can be estimated from leaf-area-based plant functional availability ($FNA_{a}$) as follows,

$\begin{matrix} LNC_{a}=PN_{p}FNA_{a}: \to\text{photosynthetic} \text{nitrogen} \\ +f_{s}(1-PN_{g})FNA_{a}: \to\text{storage nitrogen} \\ +f_{r}(1-PN_{p})PN_{g}FNA_{a}: \to\text{respiratory nitrogen} \\ +0.001LMA : \to\text{structural} \text{nitrogen} \end{matrix}$ (S4.2)

where $f_{s}$indicates the proportion of storage nitrogen that is present in leaves and $f_{r}$ is the proportion of respiratory nitrogen that is present in leaves. In the field, $f_{r}$ can be estimated from the ratio of measured plant leaf respiration to the total plant respiration. The root respiration rate is generally comparable to leaf respiration rate, while the stem respiration rate is generally very low [[1](#_ENREF_1),[2](#_ENREF_2)]. Thus, we set $f_{r}$ as 0.5 in this paper although species-specific variations would be possible. Our sensitivity analysis shows that the variation of$f_{r}$ from 0.4 to 0.6 has little effect on the relationship between *V_c,max_* and leaf nitrogen content (see Figure S1). Meanwhile, it is difficult to measure the storage nitrogen explicitly because many different form of storage nitrogen are available and Rubisco by itself could also function as storage [3,4,5]. Notice that $f_{r}$ and $f_{s}$does not affect the nitrogen allocation given a certain amount of plant functional nitrogen (i.e.,$FNA_{a}$). Instead, they only affect the estimated leaf nitrogen content given the plant functional nitrogen availability (see eq. (S4.2)). Our sensitivity analysis shows that the variation of the variation of$f_{s}$ from 0.3 to 0.8 has a strongly affect on the relationship between *V_c,max_* and leaf nitrogen content (see Figure S2) . Therefore, we treat $f_{s}$as a key unknown parameter to be fitted against data (see below for details).

With the estimated leaf nitrogen content and nitrogen allocation coefficients for functional nitrogen, we are able to estimate the nitrogen allocation at leaf level as follows,

$\begin{matrix} PNL_{str}=0.001LMA/LNC_{a} \\ PNL_{s}=f_{s}PNF_{s}FNA_{a}/LNC_{a} \\ PNL_{r}=f_{r}PNF_{r}FNA_{a}/LNC_{a} \\ PNL_{c}=PNF_{c}FNA_{a}/LNC_{a} \\ PNL_{l}=PNF_{l}FNA_{a}/LNCa\text{ } \\ PNL_{e}=PNF_{e}FNA_{a}/LNC_{a} \end{matrix}$ (S4.3)

where $PNL_{str}$,$PNL_{s}$,$PNL_{r}$, $PNL_{c}$,$PNL_{l}$and $PNL_{e}$ is the proportion of structural nitrogen, storage nitrogen, respiratory nitrogen, carboxylation nitrogen, light absorption nitrogen and electron transport nitrogen at the leaf level, respectively. See eq. (S4.1) for estimations of functional nitrogen allocation coefficients of$PNF_{s}$,$PNF_{r}$, $PNF_{c}$,$PNF_{l}$and $PNF_{e}$.

Nitrogen storage duration ($D_{ns}$) and proportion of storage nitrogen allocated to leaf (*f_s_*) are two key unknown parameters in the model. Thus, we tune $D_{ns}$ and *f_s_* to fit the model against the data. Tuning of parameter $D_{ns}$to fit the overall values of *V_c,max_* (see Figure S3), while tuning of parameter *f_s_* to fit the slope of *V_c,max_* and leaf nitrogen (see Figure S2). It can be manually tuned for simple model fitting; however, for a robust statistical analysis, we employ a Bayesian approach to estimate the two parameters. The prior distributions for the parameters are empirically specified as follows

$\begin{matrix} f_{s}\sim\text{Uniform}[0.1,0.9] \\ D_{ns}\sim\text{Uniform}[1,90] \end{matrix}$ (S4. 4)

where Uniform[$\alpha$,$\beta$] represents a uniform distribution with on the interval. With the specified prior distribution for parameters and a Gaussian distribution model for the data, Bayes’ rule is used to derive the posterior distribution for the parameters given the observed data as follows,

$\begin{matrix} f(f_{s},D_{ns}|V_{c,\max_{1}},....,V_{c,\max_{n}}) \\ \propto f(V_{c,\max_{1}},....,V_{c,\max_{n}}|f_{s},D_{ns})f(f_{s})f(D_{ns}) \\ =(\frac{1}{\sqrt{2\pi\sigma^{2}}})^{n}\exp[-\frac{1}{2\sigma^{2}}\sum_{i=1}^{n} (V_{c,\max_{i}}-V^{(p)}_{c,\max_{i}})^{2}]f(f_{s})f(D_{ns}) \end{matrix}$ (S4. 5)

where $V_{c,\max_{1}},....,V_{c,\max_{n}}$ are the measured *V_c, max_* values given a certain level of leaf nitrogen content (g/m^2^) and $V^{(p)}_{c,\max_{1}}$,…,$V^{(p)}_{c,\max_{n}}$are the predicted *V_c, max_* by our nitrogen allocation model. $\sigma$ is the standard deviation of measured *V_c,max_* minus the “true “*V_c,max_*. It is estimated in our paper using the residual stand error by fitting a linear regression for$V_{c,\max_{1}},....,V_{c,\max_{n}}$ against the leaf nitrogen content. The function $f(f_{s})$ and $f(D_{ns})$are the prior probability distribution functions defined in eq. (S4. 4). Since the posterior distribution in eq. (S4. 5) can be very difficult to derive analytically for our nitrogen allocation model, in this study, the Metropolis-Hastings approach [6,7] is used to draw samples for the parameters from the posterior distribution. Specifically, the algorithm is implemented as follows.

1. Assign initial values to $f_{s} \text{and} D_{ns}$;
2. Increase functional nitrogen availability$FNA_{a}$ from a low value (e.g., 0.5) to a high limit (e.g., 8) and for each$FNC_{a}$,  solve for $PN_{g}$,$PN_{p}$,$PN_{l}$ and $PN_{chl}$iteratively with eqs. (S1.6), (S1.7), (S1.13), and (S1.18) to estimate the nitrogen allocation coefficients within the functional nitrogen pool using eq. ();
3. Estimate area-based leaf nitrogen contents with eq. () and the nitrogen allocation coefficients within the leaf nitrogen pool with eq. () ;
4. Estimate the predicted *V_c,max_*, $V^{(p)}_{c,\max_{1}}$,…,$V^{(p)}_{c,\max_{n}}$, at the observed leaf nitrogen content (*LNC_a_*) with equation: $V_{c, max}=NUE_{c}\cdot PNL_{c}\cdot LNC_{a}$;
5. Calculate the likelihood of $f_{s} \text{and} D_{ns}$,$f(f_{s},D_{ns}|V_{c,\max_{1}},....,V_{c,\max_{n}})$, using eq. (S4. 5);
6. Propose new values for$f_{s} \text{and} D_{ns}$with a multivariate normal distribution , $f_{s} *\text{and} D_{ns}*$;
7. Calculate the likelihood of $f_{s} *\text{and} D_{ns}*$, $f(f_{s}*,D_{ns}*|V_{c,\max_{1}},....,V_{c,\max_{n}})$, based on eq. (S4. 5);
8. Draw a random sample *u* from uniform [0,1]. If

$u<\frac{f(f_{s}*,D_{ns}*|V_{c,\max_{1}},....,V_{c,\max_{n}})}{f(f_{s},D_{ns}|V_{c,\max_{1}},....,V_{c,\max_{n}})}$,

then accept the new proposed parameter values $f_{s} *\text{and} D_{ns}*$(i.e., replace $f_{s} \text{and} D_{ns}$with $f_{s} *\text{and} D_{ns}*$, repectively); otherwise stay put.

1. Repeat steps 2)-8).

It can be shown that sample drawn by the Metropolis-Hastings method will follow the posterior distribution using the fact that the Markov chain is stationary if the proposal distribution is symmetric [8]. In this study, we run a chain of 10,000 iterations and a burn size of 1000 (the initial sequence of samples that is discarded to eliminate dependence on the initial choice of parameter values). We calculate the statistics of estimated parameters using every tenth sample of the parameters (to reduce the effect of auto-correlation on sample statistics).

**Literature**

1. Wertin TM, Teskey RO (2008) Close coupling of whole-plant respiration to net photosynthesis and carbohydrates. Tree Physiology 28: 1831-1840.

2. Bolstad PV, Davis KJ, Martin J, Cook BD, Wang W (2004) Component and whole-system respiration fluxes in northern deciduous forests. Tree Physiology 24: 493-504.

3. Nordin A, Nasholm T (1997) Nitrogen storage forms in nine boreal understorey plant species. Oecologia 110: 487-492.

4. Millard P (1988) The accumulation and storage of nitrogen by herbaceous plants. Plant, Cell & Environment 11: 1-8.

5. Warren CR, Dreyer E, Adams MA (2003) Photosynthesis-Rubisco relationships in foliage of Pinus sylvestris in response to nitrogen supply and the proposed role of Rubisco and amino acids as nitrogen stores. Trees-Structure and Function 17: 359-366.

6. Metropolis N, Rosenbluth AW, Rosenbluth MN, Teller AH, Teller E (1953) Equation of state calculations by fast computing machines. Journal of Chemical Physics 21: 1087-1092.

7. Hastings WK (1970) Monte Carlo sampling methods using Markov chains and their applications. Biometrika 57: 97-109.

8. Gelman A (2004) Bayesian data analysis. Boca Raton, Fla.: Chapman & Hall/CRC. xxv, 668 p. p.
