# Supplementary material for: Toward a Mechanistic Modeling of Nitrogen Limitation on Vegetation Dynamics
Source: PLoS One. 2012 May 23;7(5):e37914. doi: 10.1371/journal.pone.0037914 (PMC3359379; doi:10.1371/journal.pone.0037914)
Supplement: Text S5 — Temperature dependence of key model parameters. (DOCX) [file pone.0037914.s005.docx]

**TEXT S5: Temperature dependence of key model parameters**

Following Collatz et al. [1] , the temperature dependence function for a kinetic parameter *k,* which can be *V_c,max_, K_c_, K_o_ and J_max_*, is calculated based on a Q_10_ function as follows,

$f_{k}(T)=Q_{10}^{(T-25)/10}$ (S5.1)

where $Q_{10}$is the rate of change of parameter increase as a consequence of increasing the [temperature](http://en.wikipedia.org/wiki/Temperature) by 10 °C and T is the leaf temperature. The $Q_{10}$ for *K_c_ and K_o_* is set to be 2.1 and 1.2, respectively. At 25^o^C, *K_c_ and K_o_* is set to be 30 and 30,000, respectively. Following the photosynthesis routine in NCAR CLM4 model [2] , the $Q_{10}$ for *V_c,max_* increases with lower temperature. Specifically,

$Q_{10}=2.4e^{\text{-0.009(T-15)}}$. (S5.2)

In view that *V_c,max_* is more sensitive to temperature than *J_max_*, the $Q_{10}$ for *J_max_* is set to be only 80% of the $Q_{10}$ for *V_c,max_* [3,4]. To simulate the enzyme denature at high temperatures, the temperature dependence function for *V_c,max_* and *J_max_* is divided by a high temperature limitation factor. Specifically,

$f_{k}(T)=\frac{Q_{10}^{(T-25)/10}}{1+e^{\frac{-220,000+703(T+273)}{R(T+273)}}}$, (S5.3)

where $R$(=8.314 *J*/*K*/*mol*) is the ideal gas constant. The Q_10_ for respiratory enzymes activities (ie.$f_{r}(T)$) is set to be same as Q_10_ for *V_c,max._*

**Literature**

1. Collatz GJ, Ball JT, Grivet C, Berry JA (1991) Physiological and environmental regulation of stomatal conductance, photosynthesis and transpiration: a model that includes a laminar boundary layer. Agricultural and Forest Meteorology 54: 107-136.

2. Oleson KW, Lawrence DM, B.Bonan G, Flanner MG, Kluzek E, et al. (2010) Technical description of version 4.0 of the Community Land Model (CLM). Boulder, CO: National Center for Atmospheric Research. 257 p.

3. Gilbert ME, Zwieniecki MA, Holbrook NM (2011) Independent variation in photosynthetic capacity and stomatal conductance leads to differences in intrinsic water use efficiency in 11 soybean genotypes before and during mild drought. Journal of Experimental Botany 62: 2875-2887.

4. Urban O, Ač A, Kalina J, Priwitzer T, Šprtová M, et al. (2007) Temperature dependences of carbon assimilation processes in four dominant species from mountain grassland ecosystem. Photosynthetica 45: 392-399.
