# Supplementary material for: Toward a Mechanistic Modeling of Nitrogen Limitation on Vegetation Dynamics
Source: PLoS One. 2012 May 23;7(5):e37914. doi: 10.1371/journal.pone.0037914 (PMC3359379; doi:10.1371/journal.pone.0037914)
Supplement: Text S6 — V c,max and Jmax standardization. (DOCX) [file pone.0037914.s006.docx]

**Text S6: *V*_c,max_ and *J_max_* standardization**

The *V_c,max_* is calculated using the photosynthesis rate (A, *µmol* CO_2_/*m*^2^/*s*) measured at relatively low leaf internal CO_2_ contention (e.g. *C_i_* < 30 pa) at saturated light conditions. Based on the Farquhar model [[1](#_ENREF_1)], *V_c,max_* is estimated by fitting the following equation to measured photosynthesis rate at different values of *C_i_*,

$$A(C_{i})=R_{c}(C_{i})V_{c,max}-R_{d}$$

where $A(C_{i})$is photosynthesis rate measured at *C_i_* (Pa) and $R_{d}$is the dark respiration rate (*µmol* CO_2_/*m*^2^/*s*). $R_{c}(C_{i})$ specifies the effects of internal CO_2_ concentration on Rubisco activity. See eq. (S3.2) in Text S3 for details of calculation for $R_{C_{i}}$. $R_{C_{i}}$depends on three parameters, *K*_c_, *K*_o_ and C*_p_*.  Different values of *K*_c_, *K*_o_ and C*_p_* and temperature dependence function can be used to estimate *V_c,max_* in different studies. To be consistent with our model, we first reconstruct the measured photosynthesis rate at different leaf internal CO_2_ concentrations. Specifically,

$$A(C_{i})^{(r)}=R_{c}(C_{i})^{(r)}V_{c,max}^{(r)}$$

where $A(C_{i})^{(r)}$is reconstructed photosynthesis rate at leaf internal CO_2_ concentrations of $C_{i}$. $V_{c,max}^{(r)}$is the reported value of$V_{c,max}$in the study of interest. $R_{c}(C_{i})^{(r)}$is the reconstructed value of$R{}_{c}(C_{i})$with eq.(S4.2) using values of *K*_c_, *K*_o_ and *C_p_* used in the study of interest. Since many studies does not report the dark respiration rate and $R_{d}$will not affect the slope of photosynthesis rate on$R_{c}(C_{i})$, we ignore the $R_{d}$ term for the reconstructed photosynthesis rates. Finally, the standardized $V_{c,max}$($V_{c,max}^{(s)}$) is calculated by fitting the following equation,

$$A(C_{i})^{(r)}=R_{c}(C_{i})^{(s)}V_{c,max}^{(s)}$$

where$R_{c}(C_{i})^{(s)}$is the value of$R_{c}(C_{i})$in eq.( S4.2) using values of *K*_c_, *K*_o_ and *C_p_* used in our current study (see Text S5 for details).

Similarly *J_max_* is calculated using the photosynthesis rate (A, *µmol* CO_2_/*m*^2^/*s*) measured at relatively high leaf internal CO_2_ contention (e.g. *C_i_* > 70 pa) at saturated light conditions. Based on the Farquhar model [[1](#_ENREF_1)], *J_max_* is estimated by fitting the following equation to measured photosynthesis rate at different values of *C_i_*,

$$A(C_{i})=R_{j}(C_{i})J_{max}-R_{d}$$

where $A(C_{i})$is photosynthesis rate measured at at *C_i_* (Pa). Similar to $V_{c,max}$ standardization, we also ignore the term $R_{d}$. $R_{j}(C_{i})$ specifies the effects of internal CO_2_ concentration on electron transport-limited rates. See eq. (S3.5) in Text S3 for details of calculation for $R_{j}(C_{i})$. $R_{j}(C_{i})$depends on three parameters, *K*_c_, *K*_o_ and C*_p_*.  Different values of *K*_c_, *K*_o_ and C*_p_* and temperature dependence function can be used to estimate *V_c,max_* in different studies. To be consistent with our model, we first reconstruct the measured photosynthesis rate at different leaf internal CO_2_ concentrations. Specifically,

$$A(C_{i})^{(r)}=R_{j}(C_{i})^{(r)}J_{max}^{(r)}$$

where $A(C_{i})^{(r)}$is reconstructed photosynthesis rate at leaf internal CO_2_ concentrations of $C_{i}$. $J_{max}^{(r)}$is the reported value of$J_{max}$in the study of interest. $R_{j}(C_{i})^{(r)}$is the reconstructed value of$R_{j}(C_{i})$with eq.(S3.5) using values of *K*_c_, *K*_o_ and *C_p_* used in the study of interest. Finally, the standardized $J_{max}$ ($J_{max}^{(s)}$) is calculated by fitting the following equation,

$$A(C_{i})^{(r)}=R_{j}(C_{i})^{(s)}J_{max}^{(s)}$$

where$R_{j}(C_{i})^{(s)}$is the value of$R_{j}(C_{i})$in eq.( S4.5) using values of *K*_c_, *K*_o_ and *C_p_* used in our current study (see Text S5 for details).

**Literature**

1. Farquhar GD, von Caemmerer S, Berry JA (1980) A biochemical model of photosynthetic CO_2_ assimilation in leaves of C_3_ species. Planta 149: 78-90.
