# Supplementary figures and images for: Toward a Mechanistic Modeling of Nitrogen Limitation on Vegetation Dynamics
Source: PLoS One. 2012 May 23;7(5):e37914. doi: 10.1371/journal.pone.0037914 (PMC3359379; doi:10.1371/journal.pone.0037914)

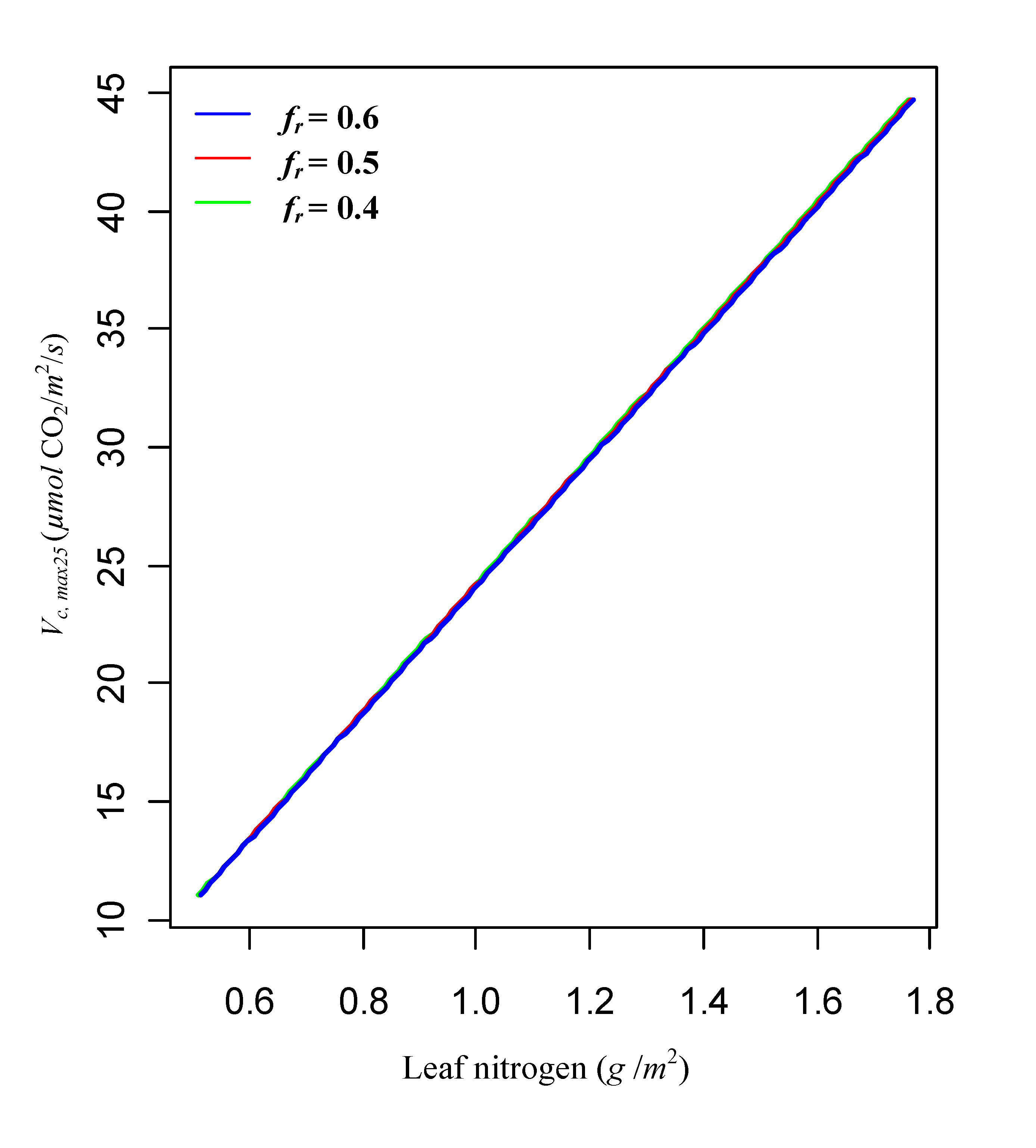

Supplement: Figure S1 — Sensitivity analysis of the relationship between Vc,max25 and leaf nitrogen to changes in proportion of respiratory nitrogen allocated to leaf ( fr ). fr increases from 0.4 to 0.6. The nitrogen storage duration is set to be 65 days. Other parameters are from test case 1 in Table 1. (TIF) [file pone.0037914.s007.tif]

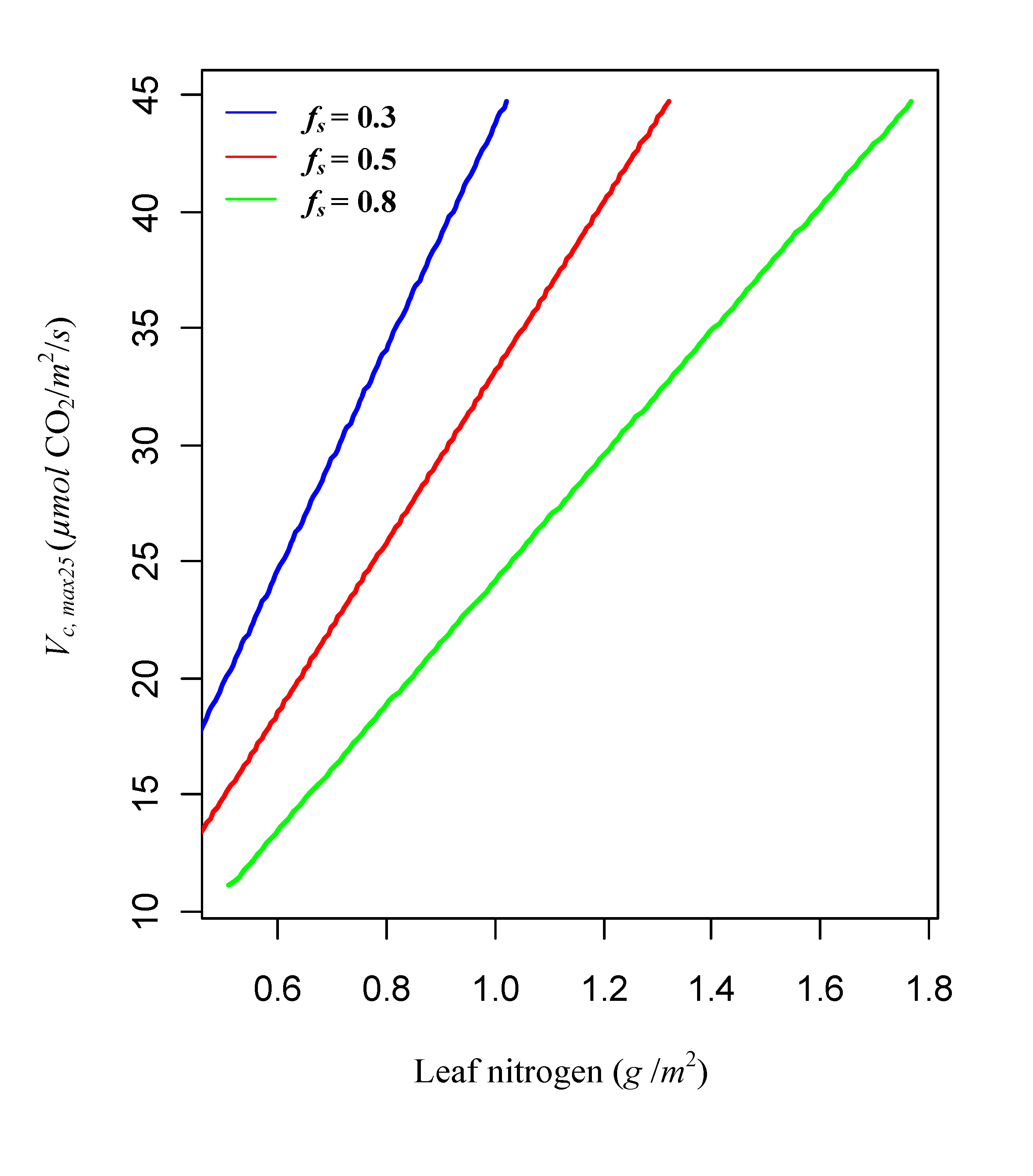

Supplement: Figure S2 — Sensitivity analysis of the relationship between Vc,max25 and leaf nitrogen to changes in proportion of storage nitrogen allocated to leaf ( fs ). fs increases from 0.3 to 0.8. The nitrogen storage duration is set to be 65 days. Other parameters are from test case 1 in Table 1. (TIF) [file pone.0037914.s008.tif]

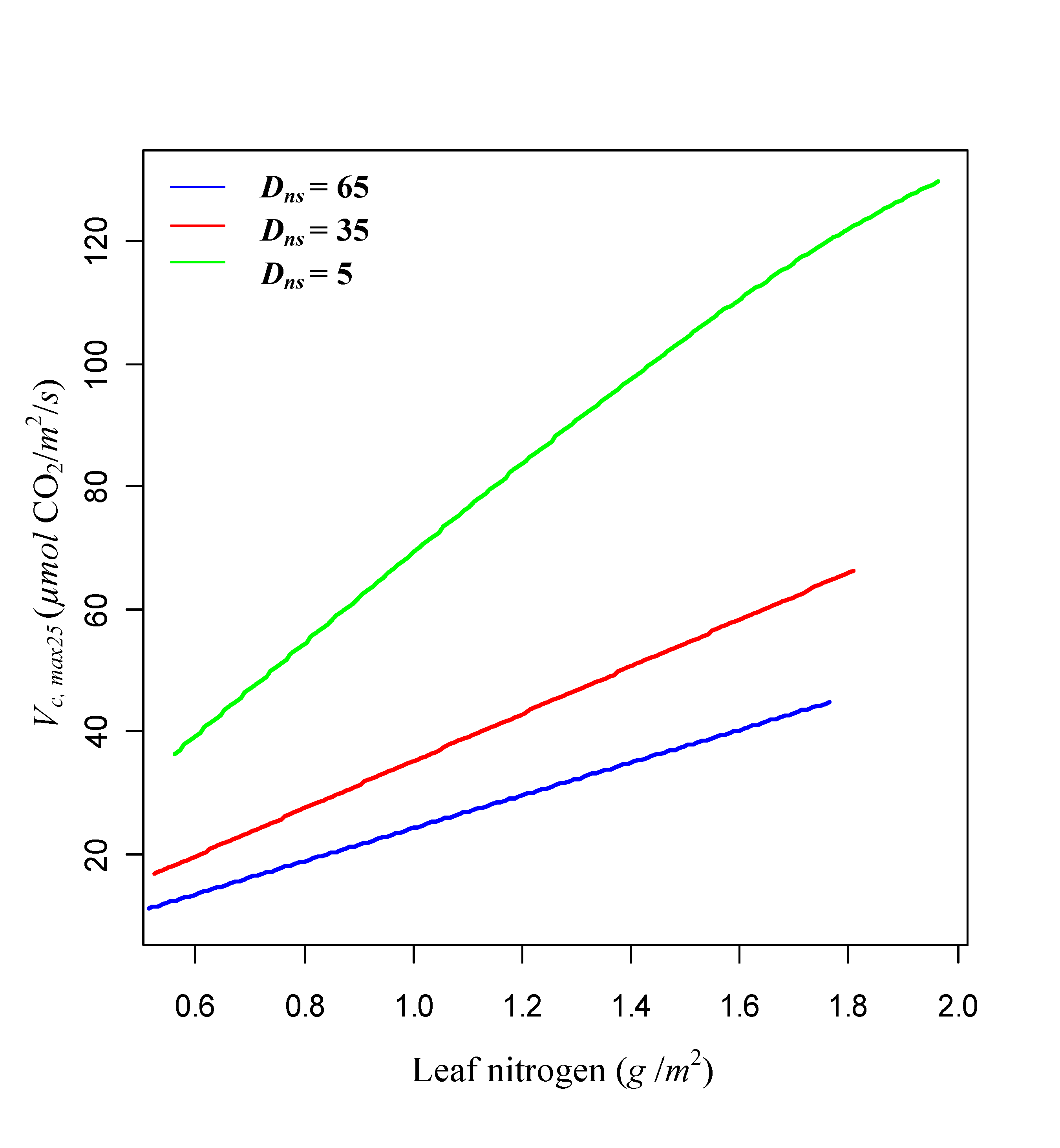

Supplement: Figure S3 — Sensitivity analysis of the relationship between Vc,max25 and leaf nitrogen to changes in nitrogen storage duration ( Dns ). Dns increases from 5 to 65. The proportion of storage nitrogen allocated to leaf (fs) is set to be 0.8. Other parameters are from test case 1 in Table 1. (TIF) [file pone.0037914.s009.tif]

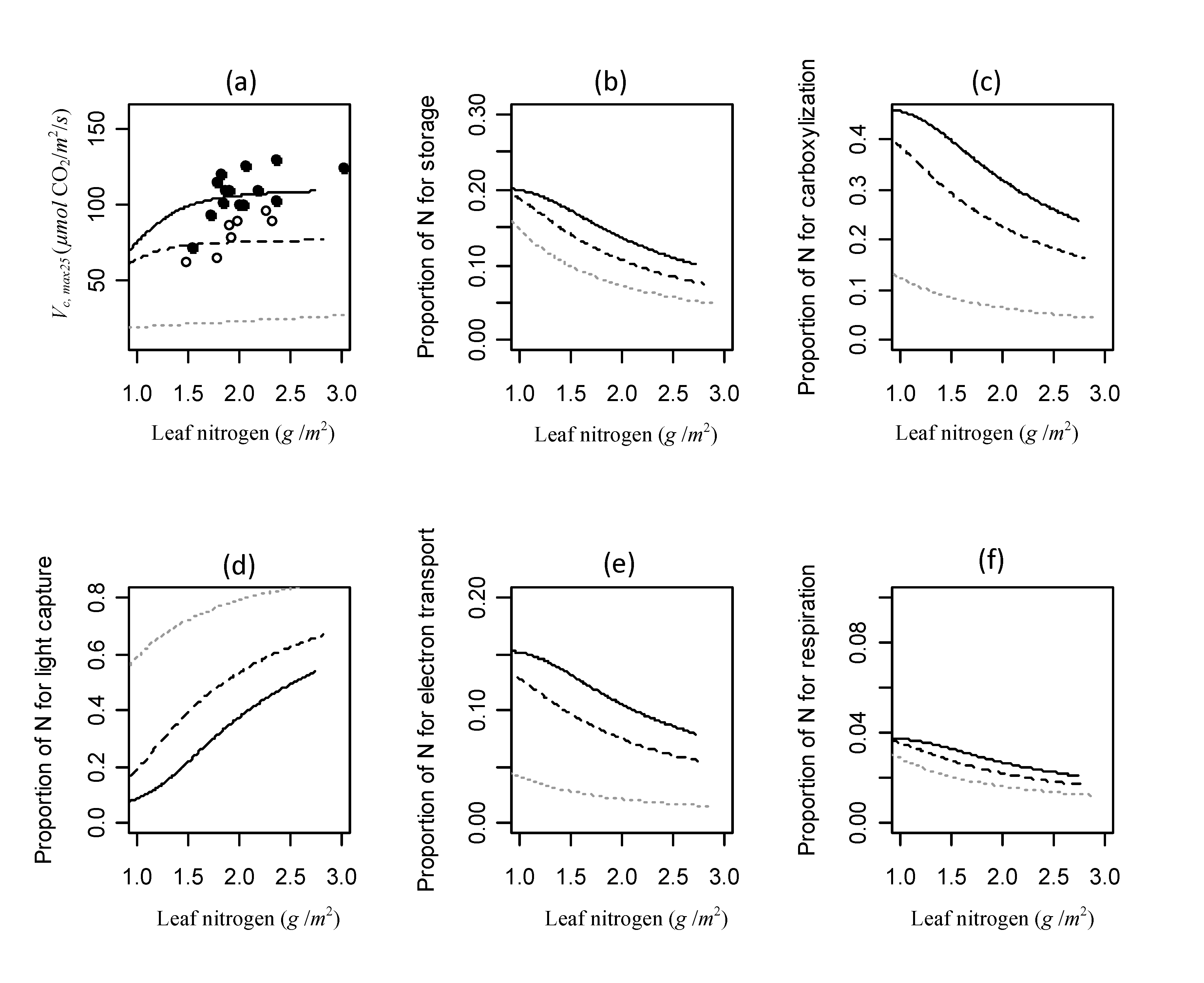

Supplement: Figure S6 — Radiation effects on leaf nitrogen allocation for test case three. In panel (a), open and closed circles represent observed Vc,max scaled to 25°C (i.e.,Vc,max25) for a Japanese plantain (Plantago asiatica) growing at a low (50 µmol photon/m 2/s) and high radiation (450 µmol photon/m 2/s) exposure, respectively. Plants in both treatments were growing at a relatively low temperature (15°C). Solid lines are predictions of Vc,max25 by the nitrogen allocation model fitted to data at a high growing temperature (30°C) and a high level of radiation(450 µmol photon/m2/s) (see filled circles in Figure S6 a). Dashed lines are predictions of Vc,max25 by the fitted nitrogen allocation model using a radiation level of 300 µmol photon /m 2/s, assume a partial acclimation. Dotted grey lines are predictions of Vc,max25 by the fitted nitrogen allocation model using a radiation level of 50 µmol/m 2/s, assuming a complete acclimation. Panels (b)–(f) show the fitted (solid lines) and predicted (dashed lines) proportion of leaf nitrogen allocated to storage, carboxylation, light capture, electron transport, and respiration, respectively. See Table 1 for main model inputs and Table 2 for fitted parameter values. (TIF) [file pone.0037914.s012.tif]
