# Supplementary material for: Toward a Mechanistic Modeling of Nitrogen Limitation on Vegetation Dynamics
Source: PLoS One. 2012 May 23;7(5):e37914. doi: 10.1371/journal.pone.0037914 (PMC3359379; doi:10.1371/journal.pone.0037914)
Supplement: Table S1 — Main model parameters. (DOCX) [file pone.0037914.s013.docx]

Table S1 Main model input and output variables

| **Category** | **Parameters** | **Descriptions** |
| --- | --- | --- |
| Parameters to be estimated directly by fitting model to *V_c,max_* data | $D_{ns}$ | Duration of time (days) that the nitrogen storage can support the current rate of carbon assimilation if nitrogen uptake were to cease altogether |
|  | $f_{s}$ | Proportion of plant storage nitrogen allocated to leaf |
| Parameters to be estimated indirectly, based on the estimation of *D_ns_* and *f_s_* | $PN_{g}$ | Proportion of nitrogen allocated for growth in functional nitrogen pool |
|  | $PN_{p}$ | Proportion of nitrogen allocated for photosynthesis in growth nitrogen pool |
|  | $PN_{l}$ | Proportion of nitrogen allocated for light harvesting in photosynthetic nitrogen pool |
|  | $PN_{chl}$ | Proportion of nitrogen allocated for light capture in light harvesting nitrogen pool |
|  | $PNF_{s}$ | Proportions of storage nitrogen within the functional nitrogen pool |
|  | $PNF_{r}$ | Proportions of respiratory nitrogen within the functional nitrogen pool |
|  | $PNF_{c}$ | Proportions of carboxylation nitrogen within the functional nitrogen pool |
|  | $PNF_{l}$ | Proportions of light capture nitrogen within the functional nitrogen pool |
|  | $PNL_{str}$ | Proportions of structural nitrogen within the leaf nitrogen pool |
|  | $PNL_{s}$ | Proportions of storage nitrogen within the leaf nitrogen pool |
|  | $PNL_{r}$ | Proportions of respiratory nitrogen within the leaf nitrogen pool |
|  | $PNL_{c}$ | Proportions of carboxylation nitrogen within the leaf nitrogen pool |
|  | $PNL_{l}$ | Proportions of light capture nitrogen within the leaf nitrogen pool |
| Model outputs | $V_{c,\max}$ | Rubisco-limited maximum rate of carboxylation rate (*µmol* CO_2_/m^2^/s) |
|  | $J_{\max}$ | Maximum electron transportation rate (*µmol* electron/m^2^/s) |
| Model input parameters | *C_v_* | Conversion factor from CO_2_ (*µmol*) to biomass (g); *C_v_* =2.4 $\times$ 10^-5^ |
|  | *C_gr_* | Proportion of gross primary production allocated for respiration; *C****_g_****_r=_0.25.* |
|  | $D_{day}$ | Day-time and nighttime length (seconds) |
|  | $D_{night}$ | Night-time length (seconds) |
|  | $f_{r}$ | Proportion of respiratory nitrogen allocated to leaf; $f_{r}$=0.5. |
|  | $SNC_{m}$ | Structural nitrogen content (*g* structural N/g plant biomass), $SNC_{m}$=0.001. |
|  | $FNA_{m}$ | Leaf-mass-based plant functional nitrogen availability (g plant functional N/g leaf), calculated as the ratio of total plant functional nitrogen to total plant leaf biomass |
|  | $FNA_{a}$ | Leaf-area-based plant functional nitrogen availability (*g* plant functional N/*m*^2^ leaf), $FNA_{a}$=$FNA_{m}\times LMA$ |
|  | $FNA_{m, ref}$ | Reference amount of plant functional nitrogen, including those in leaves, root, and sapwood, required to support the growth and maintenance of one gram leaf (*g* plant functional N/*g* leaf). |
|  | *k* | Ratio of total plant functional nitrogen to the amount of total nitrogen allocated to leaf |
|  | *K_c_* | Michealis constant for CO_2_ in Rubisco reactions (*Pa*) |
|  | *K_o_* | Competitive inhibition constant for O_2_ in Rubisco reactions (*Pa*) |
|  | LMA | Leaf mass per unit area (*g* leaf biomass/*m*^2^) |
|  | $MLNC_{m}$ | Measured mean leaf nitrogen content (*g* N/*g* leaf biomass) |
|  | $MR_{b}$ | Maintenance respiration demand per gram of functional nitrogen (*µmol* CO_2_/*g* functional nitrogen/day) |
|  | $NUE_{c}$ | Carboxylation nitrogen use efficiency (*µmol* CO_2_/ *g* N/ s). |
|  | $NUE_{J}$ | Bioenergetic electron transport nitrogen use efficiency (*µmol* electron/g N/*s*) |
|  | $NUE_{p}$ | Photosynthetic nitrogen use efficiency (*µmol* CO_2_/*g* photosynthetic N/day) |
|  | $NUE_{r}$ | Respiratory nitrogen use efficiency (*µmol* CO_2_/*g* respiratory N/day) |
|  | PAR | Photosynthetic active radiation (*µmol* photon/*g* N/*s*) |
|  | *$T_{day}$* | Day-time mean temperature (^o^C) |
|  | *$T_{night}$* | Night-time mean temperature (^o^C) |
|  | [CO_2_] | CO_2_ concentrations (*ppm*) |
|  | $\eta_{l}$ | Proportion of net carbon assimilation rate allocated to leaf |
| Model state/intermediate variables | $\alpha$ | Light absorption efficiency |
|  | $A_{n}$ | Net carbon assimilation rate (*µmol CO_2_/m^2^/day*) |
|  | *C_i_* | Internal leaf CO2 concentration (*Pa*) |
|  | $[Chl]$ | chlorophyll content (*mmol* Chl/*m*^2^ leaf ) |
|  | $FNA_{a}$ | Leaf-area-based plant functional nitrogen availability (*g* plant functional N/*m*^2^ leaf), $FNC_{a}=$LMA×$FNC_{m}$ |
|  | *J_l_* | Light harvesting rate (*µmol* electron/*m*^2^/*s*) |
|  | $LNC_{a}$ | Leaf-area-based nitrogen content (*g* N/*g* leaf biomass) |
|  | $NR_{sink}$ | Nitrogen requirement for new tissue biomass (*g* N/*g* biomass) |
|  | $PNC_{a}$ | Photosynthetic nitrogen content (*g* photosynthetic N/*m^2^* leaf)_;_ $PNC_{a}=PN_{p}PN_{g}FNC_{a}$ |
|  | $R_{c}$ | CO_2_ concentration adjustment factor for  Rubisco-limited carboxylation rate ($W_{c}$) |
|  | $R_{j}$ | CO_2_ concentration adjustment factor  for electron-transport-limited carboxylation rate ($W_{j}$) |
|  | $R_{m}$ | Maintenance respiration (*µmol* CO_2_/*m*^2^/day) |
|  | $W_{c}$ | Rubisco-limited carboxylation rate (*µmol* CO_2_/*m*^2^/s) |
|  | $W_{j}$ | Electron-limited carboxylation rate (*µmol* CO_2_/*m*^2^/s) |
|  |  |  |
